# Cost Optimisation of Individual-Based Institutional Reward Incentives for Promoting Cooperation in Finite Populations

**DOI:** 10.1007/s11538-024-01344-7

**Published:** 2024-08-05

**Authors:** M. H. Duong, C. M. Durbac, T. A. Han

**Affiliations:** 1 School of Mathematics, University of Birmingham, Birmingham, UK; 2 School of Computing, Engineering and Digital Technologies, Teesside University, Middlesbrough, UK

**Keywords:** Evolutionary game theory, Population dynamics

## Abstract

In this paper, we study the problem of cost optimisation of individual-based institutional incentives (reward, punishment, and hybrid) for guaranteeing a certain minimal level of cooperative behaviour in a well-mixed, finite population. In this scheme, the individuals in the population interact via cooperation dilemmas (Donation Game or Public Goods Game) in which institutional reward is carried out only if cooperation is not abundant enough (i.e., the number of cooperators is below a threshold $1\le t\le N-1$, where *N* is the population size); and similarly, institutional punishment is carried out only when defection is too abundant. We study analytically the cases $t=1$ for the reward incentive under the small mutation limit assumption and two different initial states, showing that the cost function is always non-decreasing. We derive the neutral drift and strong selection limits when the intensity of selection tends to zero and infinity, respectively. We numerically investigate the problem for other values of *t* and for population dynamics with arbitrary mutation rates.

## Introduction

Cooperation refers to the act of paying a cost to oneself in order to convey a benefit to somebody else. It is one of the cornerstones of human civilisation and one of the reasons for our unprecedented success as a species (Atkins et al. 2019). Organisations are constantly faced with the problem of allocating resources in a budget-effective way. This issue becomes particularly essential for institutions like local governments and the United Nations, where the optimisation of resources is crucial in facilitating cooperative endeavors. Given the paramount importance of cooperation, these organisations are tasked with strategically managing the costs associated with incentivising collective efforts (Ostrom 2005; Van Lange et al. 2014).

A well-established theoretical framework for analysing the promotion of cooperation is Evolutionary Game Theory (EGT) (Sigmund 2010), which has been used in both deterministic and stochastic settings. Using this framework, several mechanisms for promoting the evolution of cooperation have been studied including kin selection, direct reciprocity, indirect reciprocity, network reciprocity, group selection, and different forms of incentives (Nowak 2006b; Sigmund 2010; Perc et al. 2017; Rand and Nowak 2013; Van Lange et al. 2014; Xia et al. 2023; Liwen et al. 2020; Capraro and Perc 2021).

The current work focuses on institutional incentives (Sasaki et al. 2012; Sigmund et al. 2010; Wang et al. 2019; Duong and Han 2021; Cimpeanu et al. 2021; Sun et al. 2021; Van Lange et al. 2014; Gürerk et al. 2006; Góis et al. 2019; Sun et al. 2021; Liu and Chen 2022; Flores and Han 2024; Wang et al. 2024; Hua and Liu 2024), which are a plan of action involving the use of reward (i.e., increasing the payoff of cooperators), punishment (i.e., decreasing the payoff of defectors), or a combination of the two, by an external decision-maker. More precisely, we study how the aforementioned institutional incentives can be used in a cost-efficient way for maximising the levels of cooperative behaviour in a population of self-regarding individuals. In the literature, although there is a significant amount of works using agent-based numerical simulations, there are only a few papers that employ a rigorous analysis of the problem at hand (Wang et al. 2019; Han and Tran-Thanh 2018; Duong and Han 2021; Duong et al. 2023; Wang et al. 2022, 2023). The works that do use analysis employ two complementary approaches, either in a continuous setting where the evolutionary processes are modelled as a continuous dynamical system (for instance, using the replicator dynamics) (Wang et al. 2019, 2022, 2023) or in a discrete setting, in which the population dynamics is modelled as a Markov chain (Han and Tran-Thanh 2018; Duong and Han 2021; Duong et al. 2023). We review in more detail both approaches in the next paragraphs since they are most relevant to the present work.

In the discrete setting, the evolutionary process is often described by a Markov chain with an update rule (for instance, in the absence of mutation, it is an imitation process with the Fermi strategy update rule). For well-mixed finite populations and general two-player two-strategy games, the problem of promoting the evolution of cooperative behaviour with a minimum cost is formulated and numerically studied in Han and Tran-Thanh (2018). In this paper, the decision-maker may use a full-invest scheme, in which, in each generation, all cooperators are rewarded (or all defectors are punished) or an individual-based scheme, in which cooperators (defectors) are rewarded (punished, respectively) only if cooperation is not frequent enough (defection is too abundant, respectively). In both cases, the expected total cost of interference is a finite sum, over the state space of the Markov chain, of per generation costs. In Duong and Han (2021), the authors then analyse the cost function for a full-invest scheme in which individuals interact via donation games or public goods games. They prove that the cost function exhibits a phase transition when the intensity of selection varies and exactly calculate the optimal cost of the incentive for any given intensity of selection. In a more recent paper (Duong et al. 2023), similar results are obtained in the case of hybrid (mixed) reward and punishment incentives.

In the continuous setting, the evolutionary process is modelled by the replicator dynamics, which is a set of differential equations describing the evolution of the behavioural frequencies. For infinitely large, well-mixed populations, the problem of providing a minimum cost that guarantees a sufficient level of cooperation is formulated as an optimal control problem in Wang et al. (2019). By using the approach of the Hamilton–Jacobi–Bellman equation, the authors theoretically obtain the optimal reward (positive) or punishment (negative) incentive strategies with the minimal cumulative cost, respectively. Similar results for structured population are obtained in Wang et al. (2023) (for either reward or punish incentive separately) and in Wang et al. (2023) (for combined incentives), using pair approximate methods. In these papers, the decision-maker implements incentives centrally within the whole population. In Wang et al. (2022), the authors consider the same problem but using decentralised incentives, that is, in each game group, a local incentive-providing institution implements local punishment or reward incentives on group members. We also refer the reader to Wang et al. (2021) for a recent survey on this optimal control approach.

**Overview of contribution of this paper.** Following the discrete approach in Han and Tran-Thanh (2018); Duong and Han (2021); Duong et al. (2023), in this work, we rigorously study the problem of cost optimisation of institutional reward or/and punishment for maximising the levels of cooperative behaviour (or guaranteeing at least a certain level of cooperation) for well-mixed, finite populations. We focus on individual-based schemes, in which, in each generation, only when the number of cooperators/defectors is below/above a certain threshold *t* (with $1\le t< N-1$, where *N* is the population size), are they rewarded/punished, respectively (the case of the full-invest scheme $t=N-1$ has been studied in Duong and Han (2021), Duong et al. (2023)).

Analysing this problem for an arbitrary value of *t* would be very challenging due to the number of parameters involved such as the number of individuals in the population, the strength of selection, the game-specific quantities, as well as the efficiency ratios of providing the corresponding incentive. In particular, the Markov chain based evolutionary process is of order equal to the population size, which is large but finite. The calculation of the entries of the corresponding fundamental matrix, which appear in the cost function, is intricate, both analytically and computationally.

Our present work provides a rigorous analysis of this problem in the case of reward for $t = 1$. The main analytical results of the paper can be summarised as follows.

(i) We show that the cost function is always non-decreasing regardless of the values of other parameters, for two initial state assumptions, namely when the evolutionary dynamics starts either equally from the two homogeneous states or from the all-defector homogeneous state. The monotonicity of the reward cost function $E_r$ with respect to the incentive cost per capita $\theta$ highlights that, in order to achieve a higher level of cooperation, the institution needs to employ more financial resources.
(ii) We obtain the asymptotic behaviour of the cost function in the limits of neutral drift and strong selection when the intensity of selection tends to zero or infinity, respectively.

We also numerically investigate the cost function and its behaviour for other values of *t*. While the main analytical results focus on the small mutation limit $\mu \rightarrow 0$, we perform numerical simulations for the case of arbitrary mutation rates.

The rest of the paper is organised as follows. In Sect. 2, we present the model and methods. Our main results are Theorems 2 and 3 on the monotonicity of the reward cost function for $t = 1$ and Propositions 1, 2, 3, and 4 on the asymptotic behaviour (neutral drift, strong selection limits) of the reward cost function for $t = 1$, in Sect. 3. Section 4 contains the cost function (for reward, punishment, and hybrid incentives) in the case of a general mutation rate. In Sect. 5, we provide a numerical analysis of the reward cost function for both the small mutation limit and the general mutation rate cases, highlighting its qualitative behaviour. Summary and further discussions are provided in Sect. 6. Finally, Sect. 1 contains small population computations, detailed calculations for obtaining the reward cost function for $t = 2$, as well as numerical simulations for the punishment and hybrid cost functions.

## Model and Methods

In this section, we present the model and methods of the paper. We first introduce the class of games, namely cooperation dilemmas, that we are interested in throughout this work.

### Evolutionary Processes

We consider an evolutionary process of a well-mixed, finite population of *N* interacting individuals (players) and we model the finite population dynamics on an absorbing Markov chain of $(N+1)$ states, $\{S_0,..., S_N\}$, where $S_j$ represents a population with *j* cooperators (and $N-j$ defectors) (the sates $S_0$ and $S_N$ are absorbing). We employ the Fermi strategy update rule (Traulsen and Nowak 2006) stating that a player *X* with fitness $f_X$ adopts the strategy of another player *Y* with fitness $f_Y$ with a probability given by $P_{X,Y}=\left( 1 + e^{-\beta (f_Y-f_X)}\right) ^{-1}$, where $\beta$ represents the intensity of selection.

### Cooperation Dilemmas

Individuals engage with one another using one of the following one-shot (i.e., non-repeated) cooperation dilemmas: the Donation Game (DG) or its multi-player version, the Public Goods Game (PGG). Strategy wise, each player can choose to either cooperate (C) or defect (D).

Let $\Pi _C(j)$ be the average payoff of a C player (cooperator) and $\Pi _D(j)$ that of a D player (defector), in a population with *j* *C* players and $(N-j)$ *D* players. As can be seen below, the difference in payoffs $\delta = \Pi _C(j) - \Pi _D(j)$ in both games does not depend on *j*. For the two cooperation dilemmas considered in this paper, namely the Donation Games and the Public Goods Games, it is always the case that $\delta < 0$. This does not cover some weak social dilemmas such as the snowdrift game, where $\delta >0$ for some *j*, the general prisoner’s dilemma, and the collective risk game (Sun et al. 2021), where $\delta$ depends on *j*.

#### Donation Game (DG)

The Donation Game is a form of Prisoner’s Dilemma in which cooperation corresponds to offering the other player a benefit *B* at a personal cost *c*, satisfying that $B > c$. Defection means offering nothing. The payoff matrix of DG (for the row player) is given as followsDenoting $\pi _{X,Y}$ the payoff of a strategist *X* when playing with a strategist *Y* from the payoff matrix above, we obtain

$$\begin{aligned} \begin{aligned} \Pi _C(j)&=\frac{(j-1)\pi _{C,C} + (N-j)\pi _{C,D}}{N-1} = \frac{(j-1) (B-c) + (N-j) (-c)}{N-1},\\ \Pi _D(j)&=\frac{j\pi _{D,C} + (N-j-1)\pi _{D,D}}{N-1} =\frac{j B}{N-1}. \end{aligned} \end{aligned}$$

Thus,

$$\begin{aligned} \delta = \Pi _C(j) - \Pi _D(j) = -\Big (c + \frac{B}{N-1}\Big ). \end{aligned}$$

#### Public Goods Game (PGG)

In a Public Goods Game, players interact in a group of size *n*, where they decide to cooperate, contributing an amount $c > 0$ to a common pool, or to defect, contributing nothing to the pool. The total contribution in a group is multiplied by a factor *r*, where $1< r < n$ (for the PGG to be a social dilemma), which is then shared equally among all members of the group, regardless of their strategy. Intuitively, contributing nothing offers one a higher amount of money after redistribution.

The average payoffs, $\Pi _C(j)$ and $\Pi _D(j)$, are calculated based on the assumption that the groups engaging in a public goods game are given by multivariate hypergeometric sampling. Thereby, for transitions between two pure states, this reduces to sampling, without replacement, from a hypergeometric distribution. More precisely, we obtain (Hauert et al. 2007)

$$\begin{aligned} \begin{aligned} \Pi _C(j)&= \sum ^{n-1}_{i=0}\frac{\genfrac(){0.0pt}0{j-1}{i}\genfrac(){0.0pt}0{N-j}{n-1-i}}{ \genfrac(){0.0pt}0{N-1}{n-1}} \ \left( \frac{(i+1)rc}{n} - c\right) \\&=\frac{rc}{n}\left( 1 + (j-1)\frac{n-1}{N-1}\right) - c,\\ \Pi _D(j)&=\sum ^{n-1}_{i=0}\frac{\genfrac(){0.0pt}0{j}{i}\genfrac(){0.0pt}0{N-1-j}{n-1-i}}{ \genfrac(){0.0pt}0{N-1}{n-1}} \ \frac{jrc}{n} =\frac{rc(n-1)}{n(N-1)}j. \end{aligned} \end{aligned}$$

Thus,

$$\begin{aligned} \delta = \Pi _C(j) - \Pi _D(j) = -c \left( 1 - \frac{r(N-n)}{n(N-1)} \right) . \end{aligned}$$

### Cost of Institutional Incentives

To reward a cooperator (to punish a defector), the institution has to pay an amount $\theta /a$ ($\theta /b$, respectively) so that the cooperator’s (defector’s) payoff increases (decreases) by $\theta$, where $a, b > 0$ are constants representing the efficiency ratios of providing this type of incentive.

In an institutional enforcement setting, we assume that the institution has full information about the population composition or statistics at the time of decision-making. That is, given the well-mixed population setting, we assume that the number *j* of cooperators in the population is known. While reward and punishment and their usefulness as institutional incentives have been previously studied, including in Duong and Han (2021), Duong et al. (2023) which are most relevant to this work, the approach in the aforementioned papers is a ‘full-invest’ one, i.e., in each state of the evolutionary process, all cooperators (defectors) are rewarded (punished).

In the present paper, we consider investment schemes that reward (or/and punish) *t* *C* players ($N - t$ *D* players) whenever the number of cooperators in the population does not exceed a given threshold *t* (whenever the number of defectors in the population exceeds a given threshold *t*) for $1 \le t \le N-1$. The argument for this type of approach and its applicability in real life is as follows. Firstly, if cooperation is sufficiently frequent, the cooperators might survive by themselves without further need of costly incentives. Secondly, if the institution spreads their incentive budget for too many individuals, then the impact on each individual might not be enough to alter the global dynamics.

Hence, we have, for $1\le j\le t$, the cost per generation for the incentive providing institution is

1 $$\begin{aligned} \theta _j = {\left\{ \begin{array}{ll} \frac{j}{a}\theta ,\quad \text {reward incentive},\\ \frac{N-j}{b}\theta ,\quad \text {punishment incentive},\\ \min \Big (\frac{j}{a}, \frac{N-j}{b}\Big )\theta ,\quad \text {mixed incentive}, \end{array}\right. } \end{aligned}$$

while $$\theta _j= 0$$ for $$t<j\le N-1$$.

Next, we derive the total expected cost of inference over all generations. We do so for two different initial states: i) randomly commencing in the state $S_0$ or the state $S_N$ and ii) starting in the state $S_0$ (as it is more likely to find the population at the homogeneous state of all defectors than that of all cooperators).

We firstly study i), i.e., the population is equally likely to start in the homogeneous state $S_0$ (no cooperators) as well as in the homogeneous state $S_N$ (all cooperators). Let $(n_{ik})_{i,k=1}^{N-1}$ be the entries of the fundamental matrix of the absorbing Markov chain of the evolutionary process. The entries give the expected number of times the population is in the state $S_j$ if it has started in the transient state $S_i$ (Kemeny 1976). Under the above assumption, a mutant can randomly equally occur either at $S_0$ or $S_N$. Thus, the expected number of visits at state $S_j$ ($1\le j\le N-1$) is $\frac{1}{2} (n_{1j} + n_{N-1,j})$. Therefore, the expected cost of inference over all generations is given by

2 $$\begin{aligned} E(\theta )=\frac{1}{2}\sum _{j=1}^{N-1} (n_{1j}+n_{N-1,j})\theta _j=\frac{1}{2}\sum _{j=1}^t (n_{1j}+n_{N-1,j})\theta _j, \end{aligned}$$

where the second equality follows from the individual-based incentives (1).

#### Remark

We comment on our assumption that the population is equally likely to start in either homogeneous state. This assumption is reasonable when mutation is negligible and is often made in many works based on agent-based simulations (Chen and Perc 2014; Cimpeanu et al. 2023; Szolnoki and Perc Oct 2017; Han et al. 2018; Sasaki et al. 2015) (in these works, simulations end whenever the population fixates in a homogeneous state). Our model therefore encapsulates the intermediate-run dynamics, an approximation that is valid if the time-scale is long enough for one type to reach fixation, but too short for the next mutant to appear. It might thus be more practically useful for the optimisation of the institutional budget for providing incentives on an intermediate timescale.

In the most general case, when mutation is frequent, the above assumption might not be suitable. For example, if cooperators are very likely to fixate in a population of defectors, but defectors are unlikely to fixate in a population of cooperators, mutants are on average more likely to appear in the homogeneous cooperative population (that is in $$S_N$$). Similarly, if defectors are very likely to fixate in a population of cooperators, but cooperators are unlikely to fixate in a population of defectors, mutants are on average more likely to appear in $$S_0$$ rather than $$S_N$$. In general, in the long-run, the population will start at $$i = 0$$ ($$i = N$$, respectively) with probability equal to the frequency of D (C) computed at the equilibrium, $$f_D = 1/(r+1)$$ ($$f_C = r/(r+1)$$, respectively), where $$r = e^{\beta (N-1)(\delta + \theta )}$$. Thus, generally, the expected number of visits at state $$S_i$$ will be $$ f_D n_{1i} + f_C n_{N-1,i}$$. The cost function will therefore be

$$\begin{aligned} E_r(\theta ) = \sum _{j=1}^{N-1} (f_D*n_{1,j} + f_C*n_{N-1,j} )*\theta _j. \end{aligned}$$

We will study the general setting in future work.

We now compute the cost function for (ii), i.e., where the dynamics starts from the state $S_0$ with no cooperators, which could be the case in the absence of incentives. Thus, the expected cost of inference over all generations is given by

3 $$\begin{aligned} E(\theta )=\sum _{j=1}^{N-1} n_{1j}\theta _j=\sum _{j=1}^t n_{1j}\theta _j. \end{aligned}$$

#### Cooperation Frequency and Optimal Incentives

Next, we construct the problem of cost optimisation of individual-based institutional incentives (reward, punishment, and hybrid) for maximising the level (or guaranteeing at least a certain level) of cooperative behaviour.

Since the population consists of only two strategies, the fixation probabilities of a C (D) player in a homogeneous population of D (C) players when the interference scheme is carried out are, respectively, Novak (2006a)

$$\begin{aligned} \begin{aligned} \rho _{D,C}&= \left( 1+\sum _{i = 1}^{N-1} \prod _{k = 1}^i \frac{1+e^{\beta (\Pi _C(k)-\Pi _D(k) + \delta (k) \theta )}}{1+e^{-\beta (\Pi _C(k)-\Pi _D(k)+ \delta (k)\theta )}} \right) ^{-1}, \\ \rho _{C,D}&= \left( 1+\sum _{i = 1}^{N-1} \prod _{k = 1}^i \frac{1+e^{\beta (\Pi _D(k)-\Pi _C(k) - \delta (k)\theta )}}{1+e^{-\beta (\Pi _D(k)-\Pi _C(k)-\delta (k)\theta )}} \right) ^{-1}. \end{aligned} \end{aligned}$$

where $\delta (k) = 1$ if $k \le t$ and $\delta (k) = 0$ otherwise.

Computing the stationary distribution using these fixation probabilities, we obtain the frequency of cooperation

$$\begin{aligned} \frac{\rho _{D,C}}{\rho _{D,C}+\rho _{C,D}}. \end{aligned}$$

Hence, this frequency of cooperation can be maximised by maximising

4 $$\begin{aligned} \max _{\theta } \left( \rho _{D,C}/\rho _{C,D}\right) . \end{aligned}$$

The fraction in Eq. (4) can be simplified as follows (Nowak 2006b)

$$\begin{aligned} \frac{\rho _{D,C}}{\rho _{C,D}}= & {} \prod _{k = 1}^{N-1} \frac{u_{i,i-1}}{u_{i,i+1}} =\prod _{k = 1}^{N-1} \frac{1 + e^{\beta [\Pi _C(k)-\Pi _D(k) + \delta (k) \theta ]}}{1 + e^{-\beta [\Pi _C(k)-\Pi _D(k) + \delta (k)\theta ]}} \\= & {} e^{\beta \sum _{k = 1}^{N-1} \left( \Pi _C(k)-\Pi _D(k) + \delta (k)\theta \right) } \\= & {} e^{\beta [(N-1) \delta + t \theta ]}. \end{aligned}$$

In the above transformation, $u_{i,i-1}$ and $u_{i,i-1}$ are the probabilities to decrease or increase the number of C players (i.e., *i*) by one in each time step, respectively.

Under neutral selection (i.e., when $\beta = 0$), there is no need to use incentives as no player is likely to copy another player and any changes in strategy that happen are due to noise as opposed to incentives. Thus, we only consider $\beta > 0$. The goal is to ensure at least an $\omega \in [0,1]$ fraction of cooperation, i.e., $\frac{\rho _{D,C}}{\rho _{D,C}+\rho _{C,D}} \ge \omega$. Thus, it follows from the equation above that

5 $$\begin{aligned} \theta \ge \theta _0(\omega ) = \frac{1}{t}\left( \frac{1}{\beta }\log \left( \frac{\omega }{1-\omega }\right) - (N-1)\delta \right) . \end{aligned}$$

It is guaranteed that, if $\theta \ge \theta _0(\omega )$, at least an $\omega$ fraction of cooperation is expected in the long run. This condition implies that the lower bound of $\theta$ monotonically depends on $\beta$. Namely, when $\omega \ge 0.5$, it increases with $\beta$ and when $\omega < 0.5$, it decreases with $\beta$.

Bringing everything together, we obtain the following constrained mathematical minimisation problem of individual-based institutional incentives (reward, punishment, and hybrid) guaranteeing at least a certain level of cooperative behaviour:

6 $$\begin{aligned} \min _{\theta \ge \theta _0} E(\theta ), \end{aligned}$$

where $E(\theta )$ can either be the reward ($E_r(\theta )$), punishment ($E_p(\theta )$), or hybrid cost function ($E_{mix}(\theta )$).

The main aim of this paper is study the above optimisation problem. We focus on the reward incentive, in which the decision-maker rewards *j* cooperators whenever $j \le t$, as in (1) (thus, we set $a=1$ in (1) throughout the paper since it does not affect our optimisation problem). In principle, the punishment and mixed incentives cases are similar, and we only provide numerical investigations for these schemes.

For $t=1$, we analytically show that $E_r(\theta )$ is non-decreasing as a function of $\theta$ for all values of other parameters. We also establish the neutral and strong selection limits of $E_r(\theta )$, that is

$$\begin{aligned} \lim _{\beta \rightarrow 0} E_r(\theta ) \quad \text {and}\quad \lim _{\beta \rightarrow \infty } E_r(\theta ). \end{aligned}$$

For other values of *t*, we numerically investigate the properties of $E_r(\theta )$.

## Reward Incentive Under a Small Mutation Limit

We first calculate the reward cost function, $E_r(\theta )$, more explicitly. To this end, we compute the expected number of times the population contains *i* C players for $1 \le i \le N-1$. Let $U = \{u_{ik}\}_{i,k = 1}^{N-1}$ denote the transition matrix between the $N-1$ transient states, $\{S_1,..., S_{N-1}\}$. According to the Fermi update rule, the transition matrix is given as follows:

7 $$\begin{aligned} u_{i,i\pm k}= & {} 0 \qquad \text { for all } k \ge 2, \nonumber \\ u_{i,i\pm 1}= & {} \frac{N-i}{N} \frac{i}{N} \left( 1 + e^{\mp \beta [\Pi _C(i) - \Pi _D(i)+\theta _i/i]}\right) ^{-1},\nonumber \\ u_{i,i}= & {} 1 - u_{i,i+1} -u_{i,i-1}. \end{aligned}$$

For simplicity, we normalise $a=1$, and obtain (recalling that $\Pi _C(i)-\Pi _D (i)=\delta$ for all $1\le i\le N-1$, and $\theta _i/i=\frac{\theta }{a}=\theta$ for $1\le i\le t$ and zero otherwise):

$$\begin{aligned} u_{i,i\pm k}&=0 \quad \text {for}\quad k\ge 2, \\u_{i,i+1}&={\left\{ \begin{array}{ll} \frac{(N-i)i}{N^2}\Big (1+e^{-\beta (\delta +\theta )}\Big )^{-1}\quad \text {for}\quad 1\le i\le t,\\ \frac{(N-i)i}{N^2}\Big (1+e^{-\beta \delta }\Big )^{-1}\quad \text {for}\quad t< i\le N-1,\\ \end{array}\right. } \\u_{i,i-1}&={\left\{ \begin{array}{ll} \frac{(N-i)i}{N^2}\Big (1+e^{\beta (\delta +\theta )}\Big )^{-1}\quad \text {for}\quad 1\le i\le t,\\ \frac{(N-i)i}{N^2}\Big (1+e^{\beta \delta }\Big )^{-1}\quad \text {for}\quad t< i\le N-1,\\ \end{array}\right. }\\ u_{i,i}&=1-u_{i,i+1}-u_{i,i-1}. \end{aligned}$$

Next, we need to calculate the entries $n_{ik}$ of the fundamental matrix $\mathcal {N}=(n_{ik})_{i,k=1}^{N-1}= (I-U)^{-1}$. By using $\frac{1}{1+m}+\frac{1}{1+\frac{1}{m}}=1$ for $m=e^{\beta [\Pi _C(i) - \Pi _D(i)+\theta _i/i]}$, we get $u_{i,i+1}+u_{i,i-1}=\frac{N-i}{N}\frac{i}{N}$. Then, by letting $V=(I-U)$, we obtain:

8 $$\begin{aligned} v_{i,i\pm k}= & {} 0 \qquad \text { for all } k \ge 2, \nonumber \\ v_{i,i\pm 1}= & {} -\frac{N-i}{N} \frac{i}{N} \left( 1 + e^{\mp \beta [\Pi _C(i) - \Pi _D(i)+\theta _i/i]}\right) ^{-1},\nonumber \\ v_{i,i}= & {} u_{i,i+1} +u_{i,i-1}=\frac{N-i}{N}\frac{i}{N}. \end{aligned}$$

We can further write $V=W \textrm{diag}\Big \{\frac{N-1}{N}\frac{1}{N},\ldots ,\frac{N-i}{N}\frac{i}{N},\ldots , \frac{1}{N}\frac{N-1}{N}\Big \}$, where

9 $$\begin{aligned} W=\begin{pmatrix} 1&{}-a&{}&{}&{}&{}&{}\\ -c&{}1&{}-a&{}&{}&{}&{}&{}\\ &{}\ddots &{}\ddots &{}\ddots &{}&{}&{}\\ &{}&{}-c&{}1&{}-a&{}&{}&{}\\ &{}&{}&{}-d&{}1&{}-b&{}&{}&{}\\ &{}&{}&{}&{}\ddots &{}\ddots &{}\ddots &{}\\ &{}&{}&{}&{}&{}-d&{}1&{}-b\\ &{}&{}&{}&{}&{}&{}-d&{}1 \end{pmatrix}, \end{aligned}$$

with $$a:=(1+e^{-\beta (\delta +\theta )})^{-1}, \quad b:=(1+e^{-\beta \delta })^{-1}, \quad c:=(1+e^{\beta (\delta +\theta )})^{-1}, \quad d:=(1+e^{\beta \delta })^{-1}$$.

This implies that $\mathcal {N}=V^{-1}=\textrm{diag}\Big \{\frac{N^2}{N-1},\frac{N^2}{2(N-2)},\ldots ,\frac{N^2}{N-1}\Big \}W^{-1}$, and so, the fundamental matrix is $\mathcal {N}= \textrm{diag}\Big \{\frac{N^2}{N-1},\frac{N^2}{2(N-2)},\ldots ,\frac{N^2}{N-1}\Big \}(n_{ik})_{i,k=1}^{N-1}= W^{-1}$.

Therefore, the expected total cost of interference for reward in the case of the dynamics starting equally in either state $S_0$ or state $S_N$ is

10 $$\begin{aligned} E_r(\theta )&= \frac{1}{2} \sum _{j=1}^{N-1}(n_{1j} + n_{N-1,j}) \theta _j \nonumber \\&=\frac{1}{2} \sum _{j=1}^{t}(n_{1j} + n_{N-1,j}) \theta _j \nonumber \\&= \frac{N^2\theta }{2} \sum _{j=1}^{t}\frac{(W^{-1})_{1,j} + (W^{-1})_{N-1,j}}{N-j}, \end{aligned}$$

where $\theta _j = \theta j$ follows from Eq. (2) normalised with $a = 1$.

Similarly, when the population starts in the state $S_0$, we have

11 $$\begin{aligned} E_r(\theta )&= \sum _{j=1}^{N-1}n_{1j}\theta _j=\sum _{j=1}^{t}n_{1j} \theta _j=\sum _{j=1}^{t}\frac{(W^{-1})_{1,j}}{N-j}. \end{aligned}$$

The advantage of expressing the cost function in terms of the entries of the matrix *W* is that this matrix is tri-diagonal. The inverse of a tri-diagonal matrix can be theoretically computed using recursive formulae, see e.g., Huang and McColl (1997). In general, these formulae are still very hard to analytically explore. However, in the special extreme cases $t=1$ and $t=N-1$, we can explicitly obtain the entries of the inverse matrix $W^{-1}$. Therefore, we obtain analytically the explicit formula for the cost function. The case $t=N-1$ has already been studied in Duong and Han (2021), Duong et al. (2023). Thus, in this paper, we study the case $t=1$, which will be subsequently discussed in detail.

### Institutional Reward with $t = 1$ for the Equally Likely Starting State

In this section, we introduce the analytical results related to the case of institutional reward with $t = 1$, when the institution provides reward only when there is a *single* cooperator in the population. We present the cost function for this particular case together with information on its monotonicity, as well as the limits for the neutral drift and strong selection.

The reward cost function for the threshold value $t=1$ is

12 $$\begin{aligned} E_r(\theta )= \frac{N^2\theta }{2} \frac{(W^{-1})_{1,1} + (W^{-1})_{N-1,1}}{N-1}, \end{aligned}$$

obtained by substituting $t = 1$ in (10). Next, we compute explicitly the entries $(W^{-1})_{1,1}$ and $(W^{-1})_{N-1,1}$. Note that, for $t=1$, the matrix *W* is a special case of a tri-diagonal matrix of the form

$$\begin{aligned} A=\left( \begin{array}{ccccccc} b_{1} &{} c_{1} &{} &{} &{} &{} &{} \\ a_{2} &{} b_{2} &{} c_{2} &{} &{} &{} &{} \\ &{} \ddots &{} \ddots &{} \ddots &{} &{} &{} \\ &{} &{} a_{j} &{} b_{j} &{} c_{j} &{} &{} \\ &{} &{} &{} \ddots &{} \ddots &{} \ddots &{} \\ &{} &{} &{} &{} a_{n-1} &{} b_{n-1} &{} c_{n-1} \\ &{} &{} &{} &{} &{} a_{n} &{} b_{n} \end{array}\right) . \end{aligned}$$

We recall the following result from Huang and McColl (1997) that provides analytical formulae for calculating the entries of the inverse matrix $A^{-1}$. We then apply this result to calculate $(W^{-1})_{1,1}$ and $(W^{-1})_{N-1,1}$.

#### Theorem 1

(Huang and McColl (1997)) Define the second-order linear recurrences

$$\begin{aligned} z_{i}=b_{i} z_{i-1}-a_{i} c_{i-1} z_{i-2} \quad i=2,3, \ldots , n \end{aligned}$$

where $$z_{0}=1, z_{1}=b_{1}$$, and

$$\begin{aligned} y_{j}=b_{j} y_{j+1}-a_{j+1} c_{j} y_{j+2} \quad j=n-1, n-2, \ldots , 1 \end{aligned}$$

where $y_{n+1}=1, y_{n}=b_{n}$. The inverse matrix $A^{-1}=\left\{ \phi _{i, j}\right\} (1 \leqslant i, j \leqslant n)$ can be expressed as

$$\begin{aligned} \phi _{j, j}=\frac{1}{b_{j}-a_{j} c_{j-1} \frac{z_{j-2}}{z_{j-1}}-a_{j+1} c_{j} \frac{y_{j+2}}{y_{j+1}}} \end{aligned}$$

where $$j=1,2, \ldots , n, a_{1}=0, c_{n}=0$$ and

$$\begin{aligned} \phi _{i, j}= {\left\{ \begin{array}{ll}-c_{i} \frac{z_{i-1}}{z_{i}} \phi _{i+1, j} &{} i<j \\ -a_{i} \frac{y_{i+1}}{y_{i}} \phi _{i-1, j} &{} i>j.\end{array}\right. } \end{aligned}$$

#### Corollary 1

(Huang and McColl (1997)) The inverse matrix $A^{-1}=\left\{ \phi _{i, j}\right\}$ can be expressed as

$$\begin{aligned} \phi _{j, j}=\frac{1}{b_{j}-a_{j} c_{j-1} \frac{z_{j-2}}{z_{j-1}}-a_{j+1} c_{j} \frac{y_{j+2}}{y_{j+1}}}, \end{aligned}$$

where $$j=1,2, \ldots , n, \ a_{1}=0$$, $$c_{n}=0$$ and

$$\begin{aligned} \phi _{i, j}= {\left\{ \begin{array}{ll}(-1)^{j-i}\left( \prod _{k=1}^{j-i} c_{j-k}\right) \frac{z_{i-1}}{z_{j-1}} \phi _{j, j} &{} i<j \\ (-1)^{i-j}\left( \prod _{k=1}^{i-j} a_{j+k}\right) \frac{y_{i+1}}{y_{j+1}} \phi _{j, j} &{} i>j.\end{array}\right. } \end{aligned}$$

We use the above results to derive the following lemma which provides an explicit formula for the reward cost function for $t=1$.

#### Lemma 1

For $t=1$, we have

13 $$\begin{aligned} E_r(\theta ) = \frac{N^2\theta }{2(N-1)} \Big (1+d^{N-2}\frac{1}{y_2}\Big )\frac{1}{1-ad\frac{y_3}{y_2}}, \end{aligned}$$

where $y_2$ and $y_3$ are found from the following backward recursive formula:

14 $$\begin{aligned} y_{N}= & {} 1,\quad y_{N-1}=1, \quad \text {and}\quad y_{N-i} = y_{N-i+1} - (bd)y_{N-i+2},\quad \text {for}\quad \nonumber \\ i= & {} 1,\ldots , N-2. \end{aligned}$$

#### Proof

We compute the two entries $W^{-1}_{1,1}$ and $W^{-1}_{N-1,1}$ appearing in (12) using Theorem 1. We recall that

$$\begin{aligned} W=\begin{pmatrix} 1&{}-a&{}&{}&{}&{}&{}\\ -c&{}1&{}-a&{}&{}&{}&{}&{}\\ &{}\ddots &{}\ddots &{}\ddots &{}&{}&{}\\ &{}&{}-c&{}1&{}-a&{}&{}&{}\\ &{}&{}&{}-d&{}1&{}-b&{}&{}&{}\\ &{}&{}&{}&{}\ddots &{}\ddots &{}\ddots &{}\\ &{}&{}&{}&{}&{}-d&{}1&{}-b\\ &{}&{}&{}&{}&{}&{}-d&{}1 \end{pmatrix}, \end{aligned}$$

with

$$\begin{aligned} a:= & {} (1+e^{-\beta (\delta +\theta )})^{-1}, \quad b:=(1+e^{-\beta \delta })^{-1}, \quad c:=(1+e^{\beta (\delta +\theta )})^{-1}, \quad \\ d:= & {} (1+e^{\beta \delta })^{-1}. \end{aligned}$$

In particular, for $t=1$, we obtain

$$\begin{aligned} W=\begin{pmatrix} 1&{}-a&{}&{}&{}&{}&{}\\ -d&{}1&{}-b&{}&{}&{}&{}&{}\\ &{}\ddots &{}\ddots &{}\ddots &{}&{}&{}\\ &{}&{}-d&{}1&{}-b&{}&{}&{}\\ &{}&{}&{}-d&{}1&{}-b&{}&{}&{}\\ &{}&{}&{}&{}\ddots &{}\ddots &{}\ddots &{}\\ &{}&{}&{}&{}&{}-d&{}1&{}-b\\ &{}&{}&{}&{}&{}&{}-d&{}1 \end{pmatrix}. \end{aligned}$$

We apply Theorem 1, the diagonal element case, and Corollary 1, the $i>j$ case, to obtain

$$\begin{aligned} W^{-1}_{1,1}&= \frac{1}{1-ad\frac{y_3}{y_2}},\\ (W^{-1})_{N-1,1}&= (-1)^{N-2}\Big (\prod \limits _{k=1}^{N-2} a_{k+1}\Big ) \frac{y_N}{y_2}\phi _{1,1} \\&= (-1)^{N-2}\Bigg (\prod \limits _{k=1}^{N-2} a_{k+1}\Bigg )\frac{1}{y_2}\frac{1}{1-ad\frac{y_3}{y_2}} \\&= d^{N-2}\frac{1}{y_2}\frac{1}{1-ad\frac{y_3}{y_2}}. \end{aligned}$$

In the above formulae, $y_2$ and $y_3$ are found from the backward recursive formula (14). Thus, by summing up the above expressions, we obtain

$$\begin{aligned} (W^{-1})_{1,1} + (W^{-1})_{N-1,1}&= \frac{1}{1-ad\frac{y_3}{y_2}} + d^{N-2}\frac{1}{y_2}\frac{1}{1-ad\frac{y_3}{y_2}} \\&= \Big (1+d^{N-2}\frac{1}{y_2}\Big )\frac{1}{1-ad\frac{y_3}{y_2}}. \end{aligned}$$

Substituting the above into (12), we get

$$\begin{aligned} E_r(\theta )&= \frac{N^2\theta }{2(N-1)} \Big (1+d^{N-2}\frac{1}{y_2}\Big )\frac{1}{1-ad\frac{y_3}{y_2}}, \end{aligned}$$

which completes the proof of this lemma. $\square$

We now calculate explicitly $y_2$ and $y_3$ using the backward recursive formula (14):

$$\begin{aligned}&\left\{ \begin{array}{l} y_{N-1}=1, \hspace{1mm} y_{N}=1 \\ y_{j}=y_{j+1}-(b d) y_{j+2} \hspace{1mm} \text {for} \hspace{1mm} j=N-2, \ldots , 2.\\ \end{array} \right. \end{aligned}$$

For convenience, we transform the above backward recursive relation to a forward one. By applying a change of variable $\hat{y}_{j}=y_{N-j}$, we get

$$\begin{aligned}&\left\{ \begin{array}{l} \hat{y}_{0}=1, \hspace{1mm} \hat{y}_{1}=1 . \\ \hat{y}_{j}=\hat{y}_{j-1}-(b d) \hat{y}_{j-2} \hspace{1mm} \text {for} \hspace{1mm} j=2,\ldots , N . \end{array}\right. \end{aligned}$$

By induction, it follows that $\hat{y}_j$ can be written in the form

$$\begin{aligned} \hat{y}_j=\sum _{k=0}^j m_k (bd)^k. \end{aligned}$$

We observe that the coefficients of the recurrence relation follow the pattern in the table below.

**Table 1:** Pattern for the coefficients in the expansion of $\hat{y}_j$ for $j=0,1\,\ldots$, in terms of the power of (*bd*)

	$(bd)^0$	$(bd)^1$	$(bd)^2$	$(bd)^3$	$(bd)^4$	$(bd)^5$
$\hat{y}_0$	1	0	0	0	0	0
$\hat{y}_1$	1	0	0	0	0	0
$\hat{y}_2$	1	$-$1	0	0	0	0
$\hat{y}_3$	1	$-$2	0	0	0	0
$\hat{y}_4$	1	$-$3	1	0	0	0
$\hat{y}_5$	1	$-$4	3	0	0	0
$\hat{y}_6$	1	$-$5	6	$-$1	0	0
$\hat{y}_7$	1	$-$6	10	$-$4	0	0
$\hat{y}_8$	1	$-$7	15	$-$10	1	0
$\hat{y}_9$	1	$-$8	21	$-$20	5	0

We observe that the entries of Table 1 are the binomial coefficients offset by a factor of *k* for every column and are alternating in sign. This suggests that $\hat{y}_j = \sum \limits _{k=0}^{j} (-1)^k {j-k \atopwithdelims ()k}(bd)^k$. In the lemma below, we prove this is indeed the case. We also obtain another expression for $\hat{y}_j$ using the general formula of a second order homogeneous recurrence relation.

#### Lemma 2

(Recurrence relation) The following formulae hold for any $j\ge 0$

1. $$\hat{y}_j= \sum \limits _{k=0}^{\lfloor \frac{j}{2}\rfloor } (-1)^k {j-k \atopwithdelims ()k} (bd)^k$$,
2. $$\hat{y}_j = \frac{x_{1} x_{2}\left( x_{1}^{j-1}-x_{2}^{j-1}\right) -\left( x_{1}^{j}-x_{2}^{j}\right) }{x_{2}-x_{1}}$$,

where

$$\begin{aligned} x_{1,2}=\frac{1 \pm \sqrt{1-4 b d}}{2}. \end{aligned}$$

#### Proof

We prove the first statement by induction on *j*. For $j = 2$ we have $\hat{y}_2 = \hat{y}_1-(b d) \hat{y}_0 = 1 - bd$. We assume the statement holds for *j* and need to prove it for $j+1$. In fact, we have

15 $$\begin{aligned} \hat{y}_{j+1}&=\hat{y}_{j}-(b d) \hat{y}_{j-1} \nonumber \\&=\sum _{k=0}^{\lfloor \frac{j}{2}\rfloor } (-1)^k {j-k \atopwithdelims ()k}(b d)^{k}-(b d) \sum _{k=0}^{\lfloor \frac{j-1}{2}\rfloor }(-1)^k {j-1-k \atopwithdelims ()k}(b d)^{k} \nonumber \\&=\sum _{k=0}^{\lfloor \frac{j}{2}\rfloor }(-1)^k {j-k \atopwithdelims ()k}(b d)^{k}-\Bigg (\sum _{k=0}^{\lfloor \frac{j-1}{2}\rfloor } (-1)^k {j-1-k \atopwithdelims ()k}(b d)^{k+1}\Bigg ) \nonumber \\&=\sum _{k=0}^{\lfloor \frac{j}{2}\rfloor } (-1)^k {j-k \atopwithdelims ()k}(b d)^{k}-\sum _{k=1}^{\lfloor \frac{j+1}{2}\rfloor } (-1)^{k-1} {j-k \atopwithdelims ()k-1}(b d)^{k} \nonumber \\&= 1 +\sum _{k=1}^{\lfloor \frac{j}{2}\rfloor }\Bigg ((-1)^k {j-k \atopwithdelims ()k}-(-1)^{k-1} {j-k \atopwithdelims ()k-1}\Bigg )(b d)^{k} \nonumber \\&\quad - (-1)^{\lfloor \frac{j+1}{2}\rfloor - 1} {j- \lfloor \frac{j+1}{2}\rfloor \atopwithdelims ()\lfloor \frac{j+1}{2}\rfloor - 1} (bd)^{\lfloor \frac{j+1}{2}\rfloor } \nonumber \\&= 1 +\sum _{k=1}^{\lfloor \frac{j}{2}\rfloor }(-1)^k \Bigg ({j-k \atopwithdelims ()k} + {j-k \atopwithdelims ()k-1}\Bigg )(b d)^{k} \nonumber \\&\quad + (-1)^{\lfloor \frac{j+1}{2}\rfloor } {j- \lfloor \frac{j+1}{2}\rfloor \atopwithdelims ()\lfloor \frac{j+1}{2}\rfloor - 1} (bd)^{\lfloor \frac{j+1}{2}\rfloor } \nonumber \\&= 1 +\sum _{k=1}^{\lfloor \frac{j}{2}\rfloor }(-1)^k {j+1-k \atopwithdelims ()k}(b d)^{k} + (-1)^{\lfloor \frac{j+1}{2}\rfloor } {j- \lfloor \frac{j+1}{2}\rfloor \atopwithdelims ()\lfloor \frac{j+1}{2}\rfloor - 1} (bd)^{\lfloor \frac{j+1}{2}\rfloor } \nonumber \\&=\sum _{k=0}^{\lfloor \frac{j+1}{2}\rfloor } (-1)^k {j+1-k \atopwithdelims ()k}(b d)^{k} , \end{aligned}$$

where, to obtain (15), we used Pascal’s identity

$$\begin{aligned} {j-k \atopwithdelims ()k} + {j-k \atopwithdelims ()k-1} = {j+1-k \atopwithdelims ()k} \quad \text {for} \quad 1\le k \le \lfloor \frac{j}{2}\rfloor \end{aligned}$$

and

$$\begin{aligned} {j+1-\lfloor \frac{j+1}{2}\rfloor \atopwithdelims ()\lfloor \frac{j+1}{2}\rfloor } = {j - \lfloor \frac{j+1}{2}\rfloor \atopwithdelims ()\lfloor \frac{j+1}{2}\rfloor } + {j-\lfloor \frac{j+1}{2}\rfloor \atopwithdelims ()\lfloor \frac{j+1}{2}\rfloor - 1}, \end{aligned}$$

with $${j-\lfloor \frac{j+1}{2}\rfloor \atopwithdelims ()\lfloor \frac{j+1}{2}\rfloor - 1} = 0$$ as $$\lfloor \frac{j+1}{2}\rfloor - 1 > j-\lfloor \frac{j+1}{2}\rfloor $$.

Moreover, $\lfloor \frac{j-1}{2} \rfloor + 1 = n+1= \lfloor \frac{j+1}{2} \rfloor$ if $j=2n+1$ and $\lfloor \frac{j-1}{2} \rfloor + 1 = n = \lfloor \frac{j+1}{2} \rfloor$ if $j=2n$.

For the proof of the second statement, we note that $\hat{y}_{j}=\hat{y}_{j-1}-(b d) \hat{y}_{j-2}$ is a second order homogeneous recurrence relation and we solve it using the characteristic equation

$$\begin{aligned} x^{2}-x+b d=0. \end{aligned}$$

Recall $b=\frac{1}{1+e^{-\beta \delta }}$ and $d=\frac{1}{1+e^{\beta \delta }}$, so

$$\begin{aligned} b d&=\frac{1}{\left( 1+e^{\beta \delta }\right) \left( 1+e^{-\beta \delta }\right) } \\&=\frac{1}{2+e^{\beta \delta }+e^{-\beta \delta }}. \end{aligned}$$

Since $$e^{\beta \delta }+e^{-\beta \delta } \ge 2 \sqrt{e^{\beta \delta } e^{-\beta \delta }}=2$$, $$b d < \frac{1}{4}$$ $$(\text {as }\beta , \delta \ne 0)$$.

Therefore, the characteristic equation has two real solutions

$$\begin{aligned} x_{1,2}=\frac{1 \pm \sqrt{1-4 b d}}{2}. \end{aligned}$$

Thus, we can express $\hat{y}_i$ using these roots as

$$\begin{aligned} \hat{y}_{j}=r_{1} x_{1}^{j}+r_{2} x_{2}^{j}, \end{aligned}$$

where the two constants are found from the initial data:

$$\begin{aligned} \hat{y}_{0}=r_{1}+r_{2}=1 \quad \text {and}\quad \hat{y}_{1}=r_{1} x_{1}+r_{2} x_{2}=1. \end{aligned}$$

Solving the above system for $r_1, r_2$ results in

$$\begin{aligned} r_{1}=\frac{x_{2}-1}{x_{2}-x_{1}},\quad r_{2}=\frac{1-x_{1}}{x_{2}-x_{1}}. \end{aligned}$$

Hence

$$\begin{aligned} \begin{aligned} \hat{y}_{j}&=\frac{x_{2}-1}{x_{2}-x_{1}} x_{1}^{j}-\frac{x_{1}-1}{x_{2}-x_{1}} x_{2}^{j} \\&=\frac{x_{1}^{j} x_{2}-x_{1} x_{2}^{j}-\left( x_{1}^{j}-x_{2}^{j}\right) }{\left( x_{2}-x_{1}\right) } \\&=\frac{x_{1} x_{2}\left( x_{1}^{j-1}-x_{2}^{j-1}\right) -\left( x_{1}^{j}-x_{2}^{j}\right) }{x_{2}-x_{1}}. \end{aligned} \end{aligned}$$

This completes the proof of the lemma. $\square$

The following theorem, which is the main analytical result of the present paper, provides an explicit formula for the reward cost function $E_r(\theta )$ and shows that it is always non-decreasing for all parameter values.

#### Theorem 2

(Derivative and monotonicity of the cost function) $E_r'(\theta )$ is always increasing with respect to $\theta$ for all values of $N,\theta ,\beta$, where

$$\begin{aligned} E_r'(\theta )=\frac{N^2}{(2N-2)}\Big (y_2+d^{N-2}\Big )\Big (\frac{1}{y_2-ady_3}\Big )\Big [1+\frac{\theta dy_3\beta e^{-\beta (\delta +\theta ) }a^2}{y_2-ad y_3}\Big ]. \end{aligned}$$

As a consequence, the minimisation problem (6) has a unique solution

$$\begin{aligned} \min \limits _{\theta \ge \theta _0} E_r(\theta )= E_r(\theta _0). \end{aligned}$$

#### Proof

Recalling from Lemma 1 that

$$\begin{aligned} E_r(\theta )= \frac{N^2\theta }{2N-2}(y_2+d^{N-2})\Big (\frac{1}{y_2-ady_3}\Big ). \end{aligned}$$

Next, we compute the derivative of $E_r(\theta )$ with respect to $\theta$.

Note that only *a* depends on $\theta$

$$\begin{aligned} a=a(\theta )=(1+e^{-\beta (\theta +\delta )})^{-1}, \quad a'(\theta )=\frac{\beta e^{-\beta (\delta +\theta )}}{(1+e^{-\beta (\theta +\delta })^2} =\beta e^{-\beta (\delta +\theta )} a^2, \end{aligned}$$

while $b=(1+e^{-\beta \delta })^{-1}$ and $d=(1+e^{\beta \delta })^{-1}$ (and thus $y_2$ and $y_3$) do not depend on $\theta$.

Let

$$\begin{aligned} C=C(\theta )=\frac{1}{y_2-ady_3}. \end{aligned}$$

Then the derivative of *C* with respect to $\theta$ is given by

$$\begin{aligned} C'(\theta )&=\frac{dy_3 a'(\theta )}{(y_2-ad y_3)^2}\nonumber \\&=\frac{d y_3 \beta e^{-\beta (\delta +\theta )} a^2}{(y_2-ad y_3)^2}\nonumber \\&=\frac{d y_3 \beta e^{-\beta (\delta +\theta )} a^2}{(y_2-ad y_3)}C(\theta )\nonumber \\&= \frac{y_{3} \beta e^{- \beta \left( \delta + \theta \right) }}{\left( 1 + e^{- \beta \left( \delta + \theta \right) }\right) ^{2} \left( y_{2} - \frac{y_{3}}{\left( 1 + e^{- \beta \left( \delta + \theta \right) }\right) \left( 1+ e^{\beta \delta }\right) }\right) ^{2} \left( 1 + e^{\beta \delta }\right) }. \end{aligned}$$

We calculate $E'_r(\theta )$ via the product rule:

$$\begin{aligned} E_r'(\theta )&=\frac{N^2}{2N-2}(y_2+d^{N-2})\Big [C(\theta )+\theta C'(\theta )\Big ]\\&=\frac{N^2}{2N-2}(y_2+d^{N-2})\Big [1+\frac{\theta dy_3\beta e^{-\beta (\delta +\theta ) }a^2}{y_2-ad y_3}\Big ]C(\theta ) \\&= \frac{N^2}{(2N-2)}\Big (y_2+d^{N-2}\Big )\Big (\frac{1}{y_2-ady_3}\Big )\Big [1+\frac{\theta dy_3\beta e^{-\beta (\delta +\theta ) }a^2}{y_2-ad y_3}\Big ]. \end{aligned}$$

From the formula of $E_r(\theta )$, it follows that $y_2-ad y_3>0$.

We next show that $y_{2}>0$ and $y_{3}>0$. Recalling that $y_2=\hat{y}_{N-1}$ and $y_3=\hat{y}_{N-2}$, where the sequence of numbers $(\hat{y})_{j}$ is given in Lemma 2. We show that $\hat{y}_j>0$ for all $j\ge 0$.

In fact, according to the second statement of Lemma 2, we have

$$\begin{aligned} \hat{y}_j&= \frac{x_{1} x_{2}\left( x_{1}^{j-1}-x_{2}^{j-1}\right) -\left( x_{1}^{j}-x_{2}^{j}\right) }{x_{2}-x_{1}} \\&= \frac{x_1x_2(x_1-x_2)\sum \limits _{k=0}^{j-2} x_1^kx_2^{j-2-k}-(x_1-x_2)\sum \limits _{k=0}^{j-1} x_1^kx_2^{j-1-k}}{x_2-x_1} \\&= \sum \limits _{k=0}^{j-1} x_1^kx_2^{j-1-k} - \sum \limits _{k=0}^{j-2} x_1^{k+1}x_2^{j-1-k} \\&= \sum \limits _{k=0}^{j-2} x_1^kx_2^{j-1-k} + x_1^{j-1}x_2^0 - \sum \limits _{k=0}^{j-2} x_1^{k+1}x_2^{j-1-k} \\&= \sum \limits _{k=0}^{j-2} (x_1^kx_2^{j-1-k} - x_1^{k+1}x_2^{j-1-k}) + x_1^{j-1} \\&= \sum \limits _{k=0}^{j-2} x_1^kx_2^{j-1-k}(1-x_1) + x_1^{j-1} >0, \end{aligned}$$

as $x_1,x_2 \in (0,1)$. Hence $y_2, y_3>0$. Therefore, $E_r'(\theta )>0$ for all $\theta >0$. $\square$

### Asymptotic Limits

We now study the neutral drift and strong selection limits of the reward cost function $E_r(\theta )$ with $t=1$ for the equally likely initial state when the intensity of selection $\beta$ tends to 0 and to +$\infty$, respectively.

#### Proposition 1

(Neutral drift limit) It holds that

$$\begin{aligned} \lim \limits _{\beta \rightarrow 0}E_r(\theta )=\frac{N^{2} \theta }{N-1} \Bigg (P(N)+\frac{1}{2^{N-2}}\Bigg )\Big (\frac{2}{4P(N)-Q(N)}\Big ), \end{aligned}$$

where

$$\begin{aligned} P(N)&= 1+(-1)^1{N-2-1 \atopwithdelims ()1}\Big (\frac{1}{4}\Big )^1+(-1)^2{N-2-2 \atopwithdelims ()2}\Big (\frac{1}{4}\Big )^{2}+\ldots \\&\quad +(-1)^{\left\lfloor \frac{N-2}{2}\right\rfloor }{N-2-\lfloor \frac{N-2}{2}\rfloor \atopwithdelims ()\lfloor \frac{N-2}{2}\rfloor }\Big (\frac{1}{4}\Big )^{\lfloor \frac{N-2}{2}\rfloor },\\ Q(N)&= 1+(-1)^{1}{N-3-1 \atopwithdelims ()1}\Big (\frac{1}{4}\Big )^1+(-1)^{2}{N-3-2 \atopwithdelims ()2}\Big (\frac{1}{4}\Big )^{2}+\ldots \\&\quad + (-1)^{\left\lfloor \frac{N-3}{2}\right\rfloor }{N-3-\lfloor \frac{N-3}{2}\rfloor \atopwithdelims ()\lfloor \frac{N-3}{2}\rfloor }\Big (\frac{1}{4}\Big )^{\lfloor \frac{N-3}{2}\rfloor }. \end{aligned}$$

#### Proof

Recall from Lemma 1 that

$$\begin{aligned} E_r(\theta )= \frac{N^2\theta }{2N-2}(y_2+d^{N-2})\Big (\frac{1}{y_2-ady_3}\Big )=\frac{N^2\theta }{2N-2}\Big (1+\frac{d^{N-2}}{y_2}\Big )\Big (\frac{1}{1-ad\frac{y_3}{y_2}}\Big ). \end{aligned}$$

According to the first statement of Lemma 2

$$\begin{aligned} y_2&=\hat{y}_{N-2}= \sum \limits _{k=0}^{\lfloor \frac{N-2}{2}\rfloor }(-1)^{k}{N-2-k \atopwithdelims ()k}(b d)^{k},\\ y_3&=\hat{y}_{N-3}=\sum \limits _{k=0}^{\lfloor \frac{N-3}{2}\rfloor }(-1)^{k}{N-3-k \atopwithdelims ()k}(b d)^{k}. \end{aligned}$$

Note that *a*, *b*, and *d* depend on $\beta$, and therefore $y_2$ and $y_3$ also depend on $\beta$ through the product (*bd*). We have

$$\begin{aligned}&\lim \limits _{\beta \rightarrow 0} d^{N-2}=\lim \limits _{\beta \rightarrow 0}\left( \frac{1}{1+e^{\beta \delta }}\right) ^{N-2}=\frac{1}{2^{N-2}},\\&\lim \limits _{\beta \rightarrow 0} (bd)= \lim \limits _{\beta \rightarrow 0}\frac{1}{2+e^{\beta \delta }+e^{-\beta \delta }}= \frac{1}{4},\\&\lim \limits _{\beta \rightarrow 0} (ad)=\lim \limits _{\beta \rightarrow 0} \frac{1}{(1+e^{\beta \delta })(1+e^{-\beta (\theta +\delta )})}=\frac{1}{4}. \end{aligned}$$

It also follows that

$$\begin{aligned} \lim \limits _{\beta \rightarrow 0} y_2= P(N), \quad \lim \limits _{\beta \rightarrow 0} y_3= Q(N), \end{aligned}$$

where *P*(*N*) and *Q*(*N*) are given explicitly in the statement of the Proposition. Putting everything together yields

$$\begin{aligned} \lim _{\beta \rightarrow 0} E_{r}(\theta )&=\lim _{\beta \rightarrow 0} \Bigg (\frac{N^2\theta }{2N-2}\Big (1+\frac{d^{N-2}}{y_2}\Big )\Bigg (\frac{1}{1-ad\frac{y_3}{y_2}}\Bigg )\Bigg ) \\&=\frac{N^{2} \theta }{2(N-1)} \Bigg (1+\frac{\frac{1}{2^{N-2}}}{P(N)}\Bigg )\left( \frac{1 }{1-\frac{1}{4}\frac{Q(N)}{P(N)}}\right) \\&= \frac{N^{2} \theta }{N-1} \Bigg (P(N)+\frac{1}{2^{N-2}}\Bigg )\Big (\frac{2}{4P(N)-Q(N)}\Big ). \end{aligned}$$

$$\square $$

**Fig. 1:**
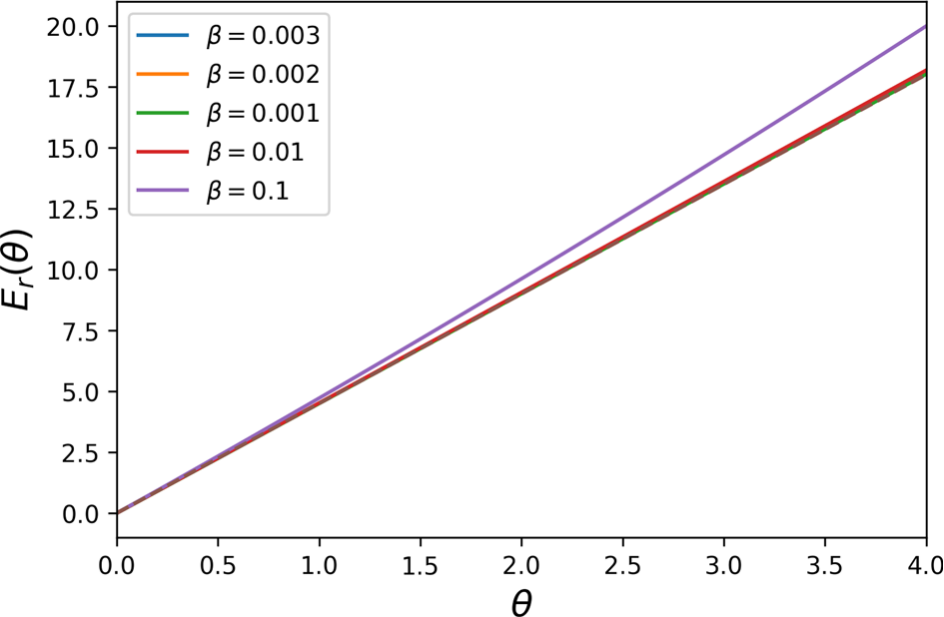
The neutral drift limit (dashed brown line) for the reward cost function $E_{r}(\theta )$ with $N = 3$, $t = 1$, and $\theta = 1$ for DG with $B = 2, c = 1$. We notice that, the more the $\beta$ value approaches 0, the closer to the limiting value the cost function $E_{r}(\theta )$ gets, in accordance to the analytical result Proposition 1

#### Proposition 2

(Strong selection limit) It holds that

$$\begin{aligned} \lim \limits _{\beta \rightarrow \infty }E_r(\theta )={\left\{ \begin{array}{ll} +\infty \quad \text {if}\quad \delta +\theta > 0,\\ \frac{2 N^2\theta }{N-1}\quad \text {if}\quad \delta +\theta = 0,\\ \frac{N^2\theta }{N-1} \quad \text {if}\quad \delta +\theta < 0. \end{array}\right. } \end{aligned}$$

#### Proof

We proceed as in the proof of the previous proposition by computing the limit of relevant quantities as $\beta \rightarrow \infty$ instead of $\beta \rightarrow 0$, noting that $\delta <0$ in our setting. We have

$$\begin{aligned}&\lim \limits _{\beta \rightarrow \infty } d^{N-2}=\lim \limits _{\beta \rightarrow \infty }\left( \frac{1}{1+e^{\beta \delta }}\right) ^{N-2}=1,\\&\lim \limits _{\beta \rightarrow \infty } (bd)= \lim \limits _{\beta \rightarrow \infty }\frac{1}{2+e^{\beta \delta }+e^{-\beta \delta }}= 0,\\&\lim \limits _{\beta \rightarrow \infty } (ad)=\lim \limits _{\beta \rightarrow \infty } \frac{1}{(1+e^{\beta \delta })(1+e^{-\beta (\theta +\delta )})}={\left\{ \begin{array}{ll} 1 \quad \text {if}\quad \delta +\theta > 0,\\ \frac{1}{2}\quad \text {if}\quad \delta +\theta = 0,\\ 0 \quad \text {if}\quad \delta +\theta < 0. \end{array}\right. } \end{aligned}$$

It also follows that

$$\begin{aligned} \lim \limits _{\beta \rightarrow \infty } y_2= 1, \quad \lim \limits _{\beta \rightarrow \infty } y_3=1. \end{aligned}$$

Putting everything together yields

$$\begin{aligned} \lim _{\beta \rightarrow 0} E_{r}(\theta )&=\lim _{\beta \rightarrow 0} \Bigg (\frac{N^2\theta }{2N-2}(1+\frac{d^{N-2}}{y_2})\Big (\frac{1}{1-ad\frac{y_3}{y_2}}\Big )\Bigg ) \\ {}&={\left\{ \begin{array}{ll} +\infty \quad \text {if}\quad \delta +\theta > 0,\\ \frac{2 N^2\theta }{N-1}\quad \text {if}\quad \delta +\theta = 0,\\ \frac{N^2\theta }{N-1} \quad \text {if}\quad \delta +\theta < 0. \end{array}\right. } \end{aligned}$$

$$\square $$

**Fig. 2:**
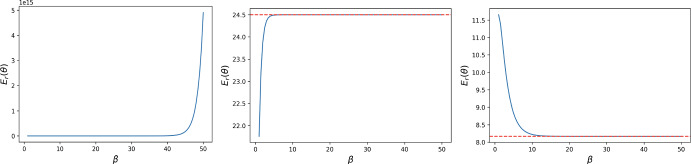
The strong selection limit (dashed red line) for the reward cost function $E_{r}(\theta )$ with $N = 7$, $t = 1$ for DG with $B = 2, c = 1$. The first image corresponds to $\delta + \theta >0$ ($\delta = -0.5$, $\theta = 1$), the middle one to $\delta + \theta = 0$ ($\delta = - 1$, $\theta = 1$), and the last one to $\delta + \theta < 0$ ($\delta$ = $-$1.5, $\theta = 1$). The numerical results are in accordance with the analytical ones in Proposition 2

### Institutional Reward with $t = 1$ for the All-Defector Starting State

In this section, we introduce the analytical results related to the case of institutional reward with $t = 1$, when the institution provides reward only when there is a *single* cooperator in the population. Differently from Sect. 3.1, here we assume that the population starts in the state $S_0$ of no cooperators (all defectors). We present the cost function for this particular case together with information on its monotonicity, as well as the limits for the neutral drift and strong selection.

The reward cost function for the threshold value $t=1$ is

16 $$\begin{aligned} E_r(\theta )= N^2\theta \frac{(W^{-1})_{1,1}}{N-1}, \end{aligned}$$

which is obtained by substituting $t = 1$ in (11). The computation of the entry $(W^{-1})_{1,1}$ is identical to the one in Sect. 3.1, that is $(W^{-1})_{1,1} = \Big ({1-ad\frac{y_3}{y_2}}\Big )$. Thus,

$$\begin{aligned} E_r(\theta ) = \frac{N^2\theta }{N-1} \Big (\frac{1}{1-ad\frac{y_3}{y_2}}\Big ). \end{aligned}$$

The following theorem, which is the counterpart of Theorem 2 in Sect. 3.1, provides an explicit formula for the reward cost function $E_r(\theta )$ and shows that it is always non-decreasing for all parameter values.

#### Theorem 3

(Derivative and monotonicity of the cost function) $E_r'(\theta )$ is always increasing with respect to $\theta$ for all values of *N*, $\theta$ and $\beta$, where

$$\begin{aligned} E_r'(\theta )=\frac{N^2}{N-1}\Big (\frac{y_2}{y_2-ady_3}\Big )\Big [1+\frac{\theta dy_3\beta e^{-\beta (\delta +\theta ) }a^2}{y_2-ad y_3}\Big ]. \end{aligned}$$

As a consequence, the minimisation problem (6) has a unique solution

$$\begin{aligned} \min \limits _{\theta \ge \theta _0} E_r(\theta )= E_r(\theta _0). \end{aligned}$$

#### Proof

We proceed like in Theorem 2, recalling from Lemma 1 that

$$\begin{aligned} E_r(\theta ) = \frac{N^2\theta }{N-1} \Big (\frac{1}{1-ad\frac{y_3}{y_2}}\Big ). \end{aligned}$$

Noting, as before, that only *a* depends on $\theta$, while *b* and *d* (and thus $y_2$ and $y_3$) do not, we let $C=C(\theta )=\frac{y_2}{y_2-ady_3}$ and compute its derivative

$$\begin{aligned} C'(\theta ) = \frac{y_{3} \beta e^{- \beta \left( \delta + \theta \right) }}{\left( 1 + e^{- \beta \left( \delta + \theta \right) }\right) ^{2} \left( y_{2} - \frac{y_{3}}{\left( 1 + e^{- \beta \left( \delta + \theta \right) }\right) \left( 1+ e^{\beta \delta }\right) }\right) ^{2} \left( 1 + e^{\beta \delta }\right) }. \end{aligned}$$

We then calculate $E'_r(\theta )$ via the product rule:

$$\begin{aligned} E_r'(\theta )&=\frac{N^2}{N-1}\Big [C(\theta )+\theta C'(\theta )\Big ]\\&=\frac{N^2}{N-1}C(\theta )\Big [1+\frac{\theta dy_3\beta e^{-\beta (\delta +\theta ) }a^2}{y_2-ad y_3}\Big ] \\&= \frac{N^2}{N-1}\Big (\frac{y_2}{y_2-ady_3}\Big )\Big [1+\frac{\theta dy_3\beta e^{-\beta (\delta +\theta ) }a^2}{y_2-ad y_3}\Big ]. \end{aligned}$$

Finally, the proof of $E_r'(\theta )>0$ for all $\theta >0$ follows closely that of Theorem 2. $\square$

### Asymptotic Limits

We now study the neutral drift and strong selection limits of the reward cost function $E_r(\theta )$ with $t=1$ for the all-defector initial state when the intensity of selection $\beta$ tends to 0 and to +$\infty$, respectively.

#### Proposition 3

(Neutral drift limit) It holds that

$$\begin{aligned} \lim \limits _{\beta \rightarrow 0}E_r(\theta )=\frac{N^{2} \theta }{N-1} \Big (\frac{4P(N)}{4P(N)-Q(N)}\Big ), \end{aligned}$$

where

$$\begin{aligned} P(N)&= 1+(-1)^1{N-2-1 \atopwithdelims ()1}\Big (\frac{1}{4}\Big )^1+(-1)^2{N-2-2 \atopwithdelims ()2}\Big (\frac{1}{4}\Big )^{2}+\ldots \\&\quad +(-1)^{\left\lfloor \frac{N-2}{2}\right\rfloor }{N-2-\lfloor \frac{N-2}{2}\rfloor \atopwithdelims ()\lfloor \frac{N-2}{2}\rfloor }\Big (\frac{1}{4}\Big )^{\lfloor \frac{N-2}{2}\rfloor },\\ Q(N)&= 1+(-1)^{1}{N-3-1 \atopwithdelims ()1}\Big (\frac{1}{4}\Big )^1+(-1)^{2}{N-3-2 \atopwithdelims ()2}\Big (\frac{1}{4}\Big )^{2}+\ldots \\&\quad + (-1)^{\left\lfloor \frac{N-3}{2}\right\rfloor }{N-3-\lfloor \frac{N-3}{2}\rfloor \atopwithdelims ()\lfloor \frac{N-3}{2}\rfloor }\Big (\frac{1}{4}\Big )^{\lfloor \frac{N-3}{2}\rfloor }. \end{aligned}$$

#### Proof

Recall that $E_r(\theta ) = \frac{N^2\theta }{N-1} \Big (\frac{1}{1-ad\frac{y_3}{y_2}}\Big )$. Following similar lines as in the proof of Proposition 1, we obtain

$$\begin{aligned} \lim _{\beta \rightarrow 0} E_{r}(\theta )&=\lim _{\beta \rightarrow 0} \Bigg (\frac{N^2\theta }{N-1}\Bigg (\frac{1}{1-ad\frac{y_3}{y_2}}\Bigg )\Bigg ) \\&=\frac{N^{2} \theta }{N-1} \left( \frac{1 }{1-\frac{1}{4}\frac{Q(N)}{P(N)}}\right) \\&= \frac{N^{2} \theta }{N-1} \Big (\frac{4P(N)}{4P(N)-Q(N)}\Big ). \end{aligned}$$

$$\square $$

**Fig. 3:**
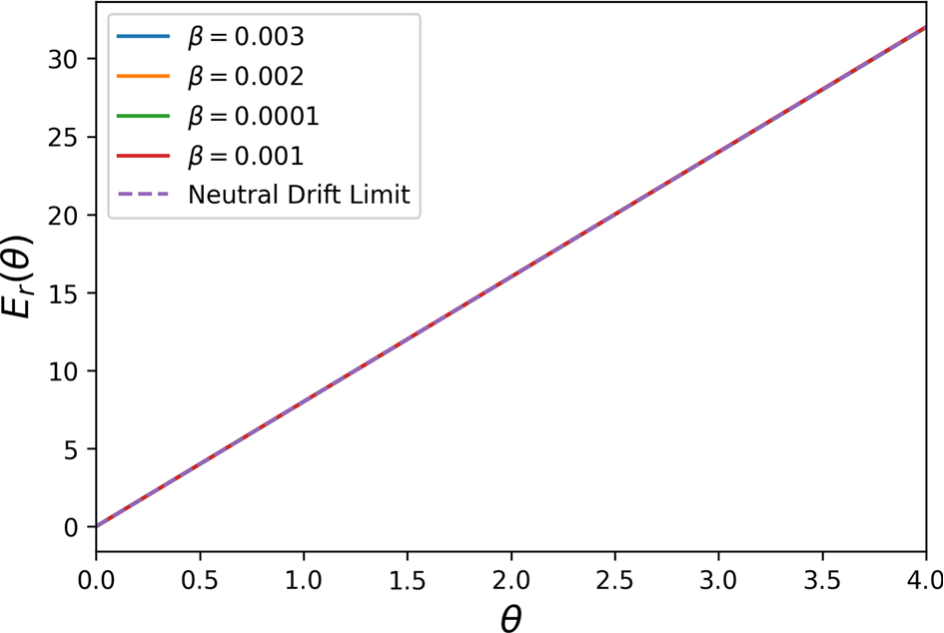
The neutral drift limit (dashed purple line) for the reward cost function $E_{r}(\theta )$ with $N = 3$, $t = 1$, and $\theta = 1$ for DG with $B = 2, c = 1$, under the assumption that the population starts with all defectors. We notice that, the closer the $\beta$ value approaches 0, the closer to the limiting value the cost function $E_{r}(\theta )$ gets, in accordance to the analytical result Proposition 3

#### Proposition 4

(Strong selection limit) It holds that

$$\begin{aligned} \lim \limits _{\beta \rightarrow \infty }E_r(\theta )={\left\{ \begin{array}{ll} +\infty \quad \text {if}\quad \delta +\theta > 0,\\ \frac{2 N^2\theta }{N-1}\quad \text {if}\quad \delta +\theta = 0,\\ \frac{N^2\theta }{N-1} \quad \text {if}\quad \delta +\theta < 0. \end{array}\right. } \end{aligned}$$

#### Proof

Recall that $E_r(\theta ) = \frac{N^2\theta }{N-1} \Big (\frac{1}{1-ad\frac{y_3}{y_2}}\Big )$. Following similar lines as in the proof of Proposition 2, we get

$$\begin{aligned} \lim _{\beta \rightarrow \infty } E_{r}(\theta )&=\lim _{\beta \rightarrow \infty } \Bigg (\frac{N^2\theta }{N-1}\Big (\frac{1}{1-ad\frac{y_3}{y_2}}\Big )\Bigg ) \\ {}&={\left\{ \begin{array}{ll} +\infty \quad \text {if}\quad \delta +\theta > 0,\\ \frac{2 N^2\theta }{N-1}\quad \text {if}\quad \delta +\theta = 0,\\ \frac{N^2\theta }{N-1} \quad \text {if}\quad \delta +\theta < 0. \end{array}\right. } \end{aligned}$$

$$\square $$

**Fig. 4:**
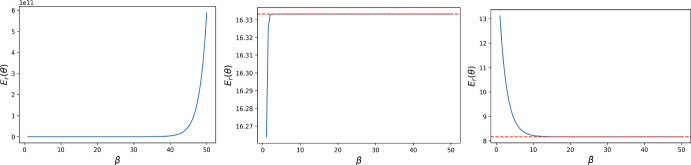
The strong selection limit (dashed red line) for the reward cost function $E_{r}(\theta )$ with $N = 7$, $t = 1$ for DG with $B = 2, c = 1$, under the assumption that the population starts with all defectors. The first image corresponds to $\delta + \theta >0$ ($\delta = -0.5$, $\theta = 1$), the middle one to $\delta + \theta = 0$ ($\delta = - 1$, $\theta = 1$), and the last one to $\delta + \theta < 0$ ($\delta$ = $-$1.5, $\theta = 1$). The numerical results are in accordance with the analytical one in Proposition 4

### Institutional Reward with $t = 2$

We compute the reward cost function for $t = 2$, i.e., when the institution provide rewards only if there are at most *two* cooperators in the population, under both starting states.

For $$t=2$$,

$$\begin{aligned} W=\begin{pmatrix} 1&{}-a&{}&{}&{}&{}&{}\\ -c&{}1&{}-a&{}&{}&{}&{}&{}\\ &{}-d&{}1&{}-b&{}&{}&{}\\ &{}&{}\ddots &{}\ddots &{}\ddots &{}\\ &{}&{}&{}-d&{}1&{}-b&{}&{}&{}\\ &{}&{}&{}&{}-d&{}1&{}-b&{}&{}&{}\\ &{}&{}&{}&{}&{}\ddots &{}\ddots &{}\ddots &{}\\ &{}&{}&{}&{}&{}&{}-d&{}1&{}-b\\ &{}&{}&{}&{}&{}&{}&{}-d&{}1 \end{pmatrix}. \end{aligned}$$

Therefore, for the equally likely starting state, by using Eq. (10), we get:

17 $$\begin{aligned} E_r(\theta )&=\frac{N^{2} \theta }{2} \sum _{j=1}^{2} \frac{\left( W^{-1}\right) _{1, j}+\left( W^{-1}\right) _{N-1, j}}{N-j} \nonumber \\&=\frac{N^{2} \theta }{2}\left[ \left( \frac{\left( W^{-1}\right) _ {1,1}+\left( W^{-1}\right) _{N-1,1}}{N-1}\right) +\left( \frac{\left( W^{-1}\right) _{1,2} +\left( W^{-1}\right) _{N-1,2}}{N-2}\right) \right] . \end{aligned}$$

To obtain a simplified version of Eq. (17), we need to compute $(W^{-1})_{1,1}$, $(W^{-1})_{N-1,1}$, $(W^{-1})_{1,2}$, $(W^{-1})_{N-1,2}$. We apply Theorem 1, the diagonal element case, and Corollary 1, both cases, to obtain:

$$\begin{aligned} (W^{-1}_{1,1})&= \frac{1}{1-ac\frac{y_3}{y_2}},\\ (W^{-1})_{N-1,1}&= c d^{N-3} \frac{1}{y_{2}} \frac{1}{1-a c \frac{y_{3}}{y_{2}}},\\ (W^{-1})_{1,2}&= a \frac{1}{1-a c-a d\frac{y_4}{y_3}},\\ (W^{-1})_{N-1,2}&= d^{N-3}\frac{1}{y_{3}} \frac{1}{1-a c-a d \frac{y_4}{y_3}}. \end{aligned}$$

See Sect. 1 for detailed computations of the *W* entries.

Substituting the *W* values in Eq. (17) yields:

$$\begin{aligned} E_r(\theta )&=\frac{N^{2} \theta }{2}\left( \frac{\frac{1}{1-a c \frac{y_{3}}{y_{2}}}+c d^{N-3} \frac{1}{y_{2}} \frac{1}{1-a c \frac{y_3}{y_2}}}{N-1}+\frac{a\frac{1}{1-ac-ad\frac{y_4}{y_3}}+d^{N-3}\frac{1}{y_3}\frac{1}{1-ac-ad\frac{y_4}{y_3}}}{N-2}\right) \\&=\frac{N^{2} \theta }{2}\left[ \frac{\frac{1}{1-a c \frac{y_{3}}{y_{2}}}\left( 1+c d^{N-3} \cdot \frac{1}{y_{2}}\right) }{N-1} + \frac{\frac{1}{1-a c-a d \frac{y_{4}}{y_{3}}}\left( a+d^{N-3} \cdot \frac{1}{y_{3}}\right) }{N-2}\right] . \end{aligned}$$

For the all-defector starting state, we have:

18 $$\begin{aligned} E_r(\theta )&=\sum _{j=1}^{2} \frac{\left( W^{-1}\right) _{1, j}}{N-j} =\frac{W^{-1}_{1,1}}{N-1}+\frac{W^{-1}_{1,2}}{N-2}. \end{aligned}$$

By substituting the *W* values in Eq. (18), we get:

$$\begin{aligned} E_r(\theta )=\frac{\frac{1}{1-a c \frac{y_{3}}{y_{2}}}}{N-1}+\frac{a\frac{1}{1-ac-ad\frac{y_4}{y_3}}}{N-2}. \end{aligned}$$

## Institutional Incentives Under General Starting State and Mutation

In this section, we present the cost function $E(\theta )$ for the case of general mutation rates. Indeed, for an arbitrary mutation rate $\mu$, the transition probabilities change as follows.

$$\begin{aligned} \begin{aligned} u_{i,i\pm k}&= 0 \qquad \text { for all } k \ge 2, \\ u_{i,i+1}&= \frac{N-i}{N}\left( \mu + (1-\mu )\frac{i}{N} \left( 1 + e^{-\beta [\Pi _C(i) - \Pi _D(i)+\theta _i/i]}\right) ^{-1}\right) ,\\ u_{i,i-1}&= \frac{i}{N}\left( \mu + (1-\mu )\frac{N-i}{N} \left( 1 + e^{\beta [\Pi _C(i) - \Pi _D(i)+\theta _i/i]}\right) ^{-1}\right) ,\\ u_{i,i}&= 1 - u_{i,i+1} -u_{i,i-1}. \end{aligned} \end{aligned}$$

Thus, the expected cost function $E(\theta )$ given the initial state of the population being $S_i$ (i.e. with *i* cooperators and $N-i$ defectors) is

19 $$\begin{aligned} E^i(\theta ) = \sum _{j=0}^{N} n_{ij} \theta _j, \end{aligned}$$

where $n_{i;j}$ is obtained from $\mathcal {N}=(n_{ij})_{i,j=0}^{N}= (I-U)^{-1}$ and

20 $$\begin{aligned} \theta _j = {\left\{ \begin{array}{ll} \frac{j}{a}\theta ,\quad \text {reward incentive},\\ \frac{N-j}{b}\theta ,\quad \text {punishment incentive},\\ \min \Big (\frac{j}{a}, \frac{N-j}{b}\Big )\theta ,\quad \text {mixed incentive}. \end{array}\right. } \end{aligned}$$

Depending on the incentive $\theta _j$ selected, $E^i(\theta )$ could become either $E^i_r(\theta )$, $E^i_p(\theta )$, or $E^i_{mix}(\theta )$. In Sect. 5.2, we perform numerical simulations on the reward cost function $E^i_r(\theta )$ for various starting points.

**Fig. 5:**
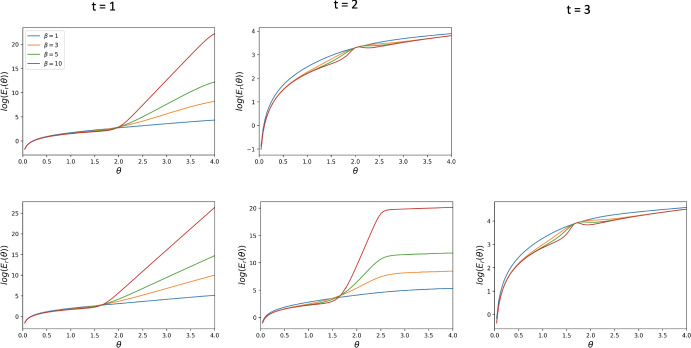
Behaviour of the reward cost function $$E_{r}(\theta )$$, for different thresholds *t* and strengths of selection $$\beta $$, for DG with $$B = 2, c = 1$$. The first row corresponds to $$N = 3$$ and the second one to $$N = 4$$. The leftmost column corresponds to $$t = 1$$, the middle one to $$t = 2 $$, the rightmost one to $$t = 3$$. This is for the assumption that the population is equally likely to start in the homogeneous state $$S_0$$ as well as in the homogeneous state $$S_N$$

**Fig. 6:**
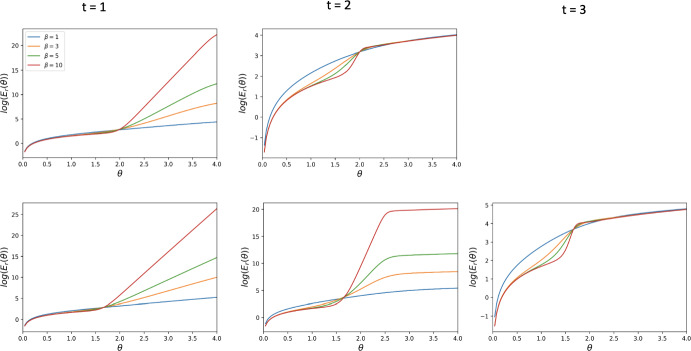
Behaviour of the reward cost function $$E_{r}(\theta )$$, for different thresholds *t* and strengths of selection $$\beta $$, for DG with $$B = 2, c = 1$$. The first row corresponds to $$N = 3$$ and the second one to $$N = 4$$. The leftmost column corresponds to $$t = 1$$, the middle one to $$t = 2 $$, the rightmost one to $$t = 3$$. This is for the assumption that the population is expected to start with all defectors, i.e., in state $$S_0$$

## Numerical Analysis

In this section, we present the results of our numerical analysis, coded in Python 3.10. We employ a logarithmic scale to respond to skewness of larger values.

### Behaviour of the Cost Functions Under Small Mutation with Different Initial States

In Figs. 5 and 6, we plot the reward cost function for DG for the two initial state assumptions: the dynamics randomly commencing in the state $S_0$ or the state $S_N$ and it starting in the state $S_0$. Figures 10 and 11 in Appendix, show the behaviour of the reward cost function for PGG, while Figs. 12 and 13 show that of the punishment cost function for DG. In Figs. 14 and 15, we plot the behaviour of the hybrid cost function for DG. The numerical simulations concern some small values of *N* and various values of the other parameters. From these plots, we observe that the cost functions $E_r(\theta )$, $E_p(\theta )$ and $E_{mix}(\theta )$ are increasing for all $1 \le t < N-1$ and become non-monotonic for $t = N-1$, exhibiting a phase transition. This behaviour is dependant on changes in the strength of selection, $\beta$. The larger this parameter is, the more pronounced the non-monotonic behaviour of the function becomes. The aforementioned behaviour is robust to changes in the game-specific values such as *B*, *c*, *r*, *n*.

### Behaviour of the Reward Cost Function Under General Mutation with Various Starting Points

**Fig. 7:**
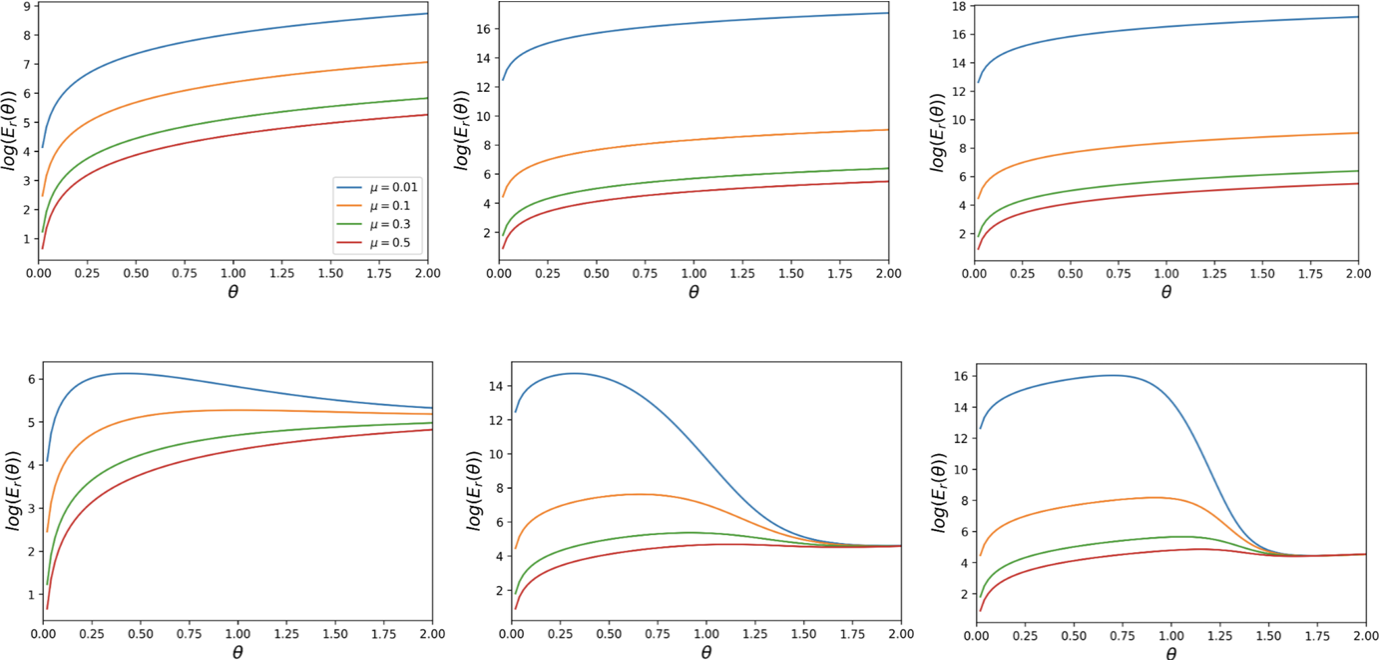
Behaviour of the reward cost function $E_r(\theta )$, for $N = 6$, $t = 1, 5$, and strengths of selection $\beta = 1, 5, 10$, for DG with $B = 2, c = 1$. The first row corresponds to $t = 1$, while the second one to $t = 5$ $(N-1)$. The first column corresponds to $\beta = 1$, the middle one to $\beta = 5$, and the last one to $\beta = 10$. The behaviour of the reward cost function for PGG is similar. This is for a general mutation rate $\mu$. The starting point is $S_0$

**Fig. 8:**
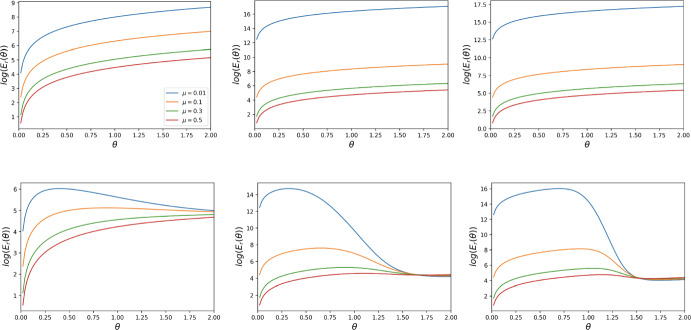
Behaviour of the reward cost function $E_r(\theta )$, for $N = 6$, $t = 1, 5$, and strengths of selection $\beta = 1, 5, 10$, for DG with $B = 2, c = 1$. The first row corresponds to $t = 1$, while the second one to $t = 5$ $(N-1)$. The first column corresponds to $\beta = 1$, the middle one to $\beta = 5$, and the last one to $\beta = 10$. The behaviour of the reward cost function for PGG is similar. This is for a general mutation rate $\mu$. The starting point is $S_{\frac{N}{2}}$

**Fig. 9:**
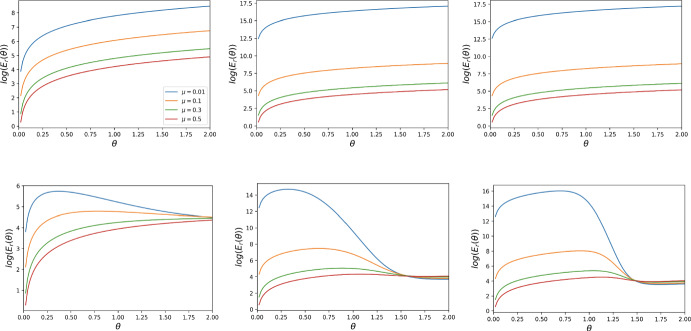
Behaviour of the reward cost function $E_r(\theta )$, for $N = 6$, $t = 1, 5$, and strengths of selection $\beta = 1, 5, 10$, for DG with $B = 2, c = 1$. The first row corresponds to $t = 1$, while the second one to $t = 19$ $(N-1)$. The first column corresponds to $\beta = 1$, the middle one to $\beta = 5$, and the last one to $\beta = 10$. The behaviour of the reward cost function for PGG is similar. This is for a general mutation rate $\mu$. The starting point is $S_{N-2}$

**Fig. 10:**
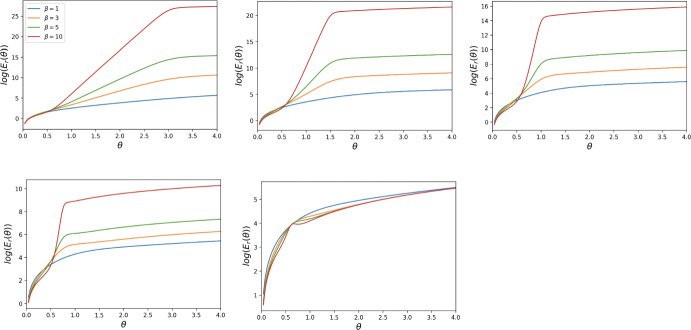
Behaviour of the reward cost function $E_{r}(\theta )$, for different thresholds *t* and strengths of selection $\beta$, for PGG with $N = 6, c = 1, r = 2, n = 3$. The first row corresponds to $t = 1$, $t = 2$, and $t = 3$, while the second one to $t = 4$ and $t = 5$. This is for the assumption that the population is equally likely to start in the homogeneous state $S_0$ as well as in the homogeneous state $S_N$

**Fig. 11:**
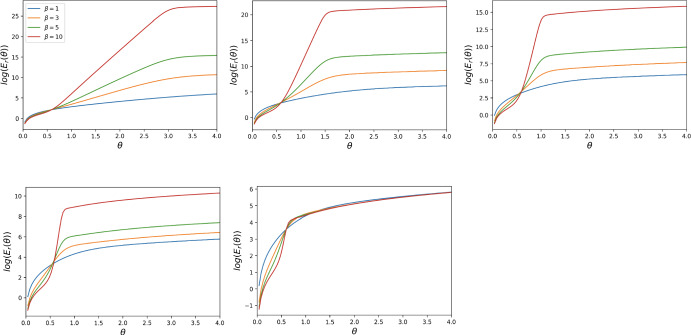
Behaviour of the reward cost function $E_{r}(\theta )$, for different thresholds *t* and strengths of selection $\beta$, for PGG with $N = 6, c = 1, r = 2, n = 3$. The first row corresponds to $t = 1$, $t = 2$, and $t = 3$, while the second one to $t = 4$ and $t = 5$. This is for the assumption that the population is expected to start with all defectors, i.e., in state $S_0$

**Fig. 12:**
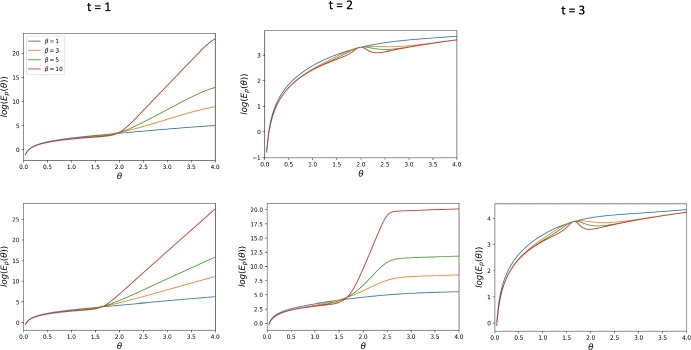
Behaviour of the punishment cost function $$E_{p}(\theta )$$, for different thresholds *t* and strengths of selection $$\beta $$, for DG with $$B = 2, c = 1$$. The first row corresponds to $$N = 3$$ and the second one to $$N = 4$$. The leftmost column corresponds to $$t = 1$$, the middle one to $$t = 2 $$, the rightmost one to $$t = 3$$. The behaviour of the cost function for PGG is similar. This is for the assumption that the population is equally likely to start in the homogeneous state $$S_0$$ as well as in the homogeneous state $$S_N$$

**Fig. 13:**
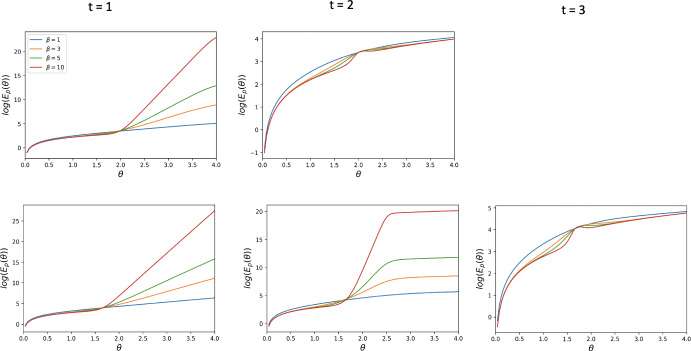
Behaviour of the punishment cost function $$E_{p}(\theta )$$, for different thresholds *t* and strengths of selection $$\beta $$, for DG with $$B = 2, c = 1$$. The first row corresponds to $$N = 3$$ and the second one to $$N = 4$$. The leftmost column corresponds to $$t = 1$$, the middle one to $$t = 2 $$, the rightmost one to $$t = 3$$. The behaviour of the cost function for PGG is similar. This is for the assumption that the population is expected to start with all defectors, i.e., in state $$S_0$$

**Fig. 14:**
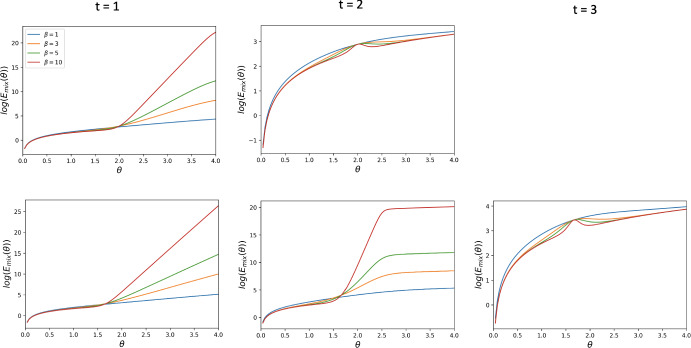
Behaviour of the hybrid incentive cost function (mix of reward and punishment) $$E_{mix}(\theta )$$ (where $$a = 1, b = 1$$), for different thresholds *t* and strengths of selection $$\beta $$, for DG with $$B = 2, c = 1$$. The first row corresponds to $$N = 3$$ and the second one to $$N = 4$$. The leftmost column corresponds to $$t = 1$$, the middle one to $$t = 2 $$, the rightmost one to $$t = 3$$. The behaviour of the cost function for PGG is similar. This is for the assumption that the population is equally likely to start in the homogeneous state $$S_0$$ as well as in the homogeneous state $$S_N$$

**Fig. 15:**
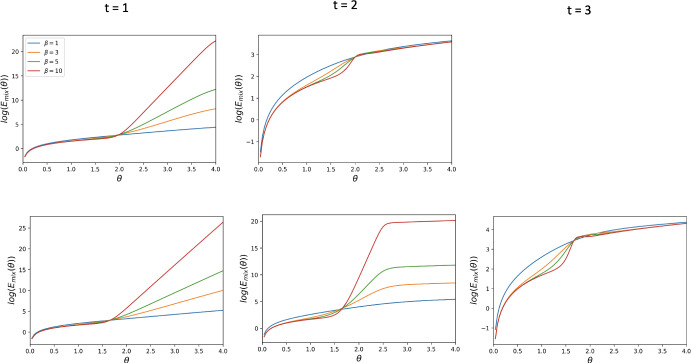
Behaviour of the hybrid incentive cost function (mix of reward and punishment) $$E_{mix}(\theta )$$ (where $$a = 1, b = 1$$), for different thresholds *t* and strengths of selection $$\beta $$, for DG with $$B = 2, c = 1$$. The first row corresponds to $$N = 3$$ and the second one to $$N = 4$$. The leftmost column corresponds to $$t = 1$$, the middle one to $$t = 2 $$, the rightmost one to $$t = 3$$. The behaviour of the cost function for PGG is similar. This is for the assumption that the population is expected to start with all defectors, i.e., in state $$S_0$$

In Figs. 7, 8 and 9, we plot the reward cost function (as a function of the per capital cost $\theta )$ for the evolutionary processes with general mutation and different starting states $S_0, S_{\frac{N}{2}}, S_{N-1}$ for $t=1$, $t=N-1$ (for some *N*), and various values of other parameters. These figures clearly show the strong and non-trivial impact of the threshold *t*, the mutation rate $\mu$, and the strength of selection $\beta$ on the cost function. We observe that for $t=1$, the cost function is decreasing with respect to $\mu$. For $t=N-1$, the function is also decreasing with respect to $\mu$ for sufficiently small $\beta$, but for sufficiently large $\beta$ it behaves much more complicatedly. To be comparable to the paper’s main analysis using the small mutation approach, we assume that the mutation rate is small enough that the cost is calculated until a homogeneous state is reached. This is in line with most previous simulation works on incentive optimisation (see again the Remark).

### Phase Transition: Change in the Qualitative Behaviour of the Cost Function

Theorems 2 and 3 show that the reward cost function for $t=1$ is always non-decreasing for all values of the intensity of selection $\beta$. In Duong and Han (2021), it is shown that, for $t=N-1$, $E_r(\theta )$ and $E_p(\theta )$ are non-decreasing when $\beta$ is sufficiently small, but are not monotonic when $\beta$ is large enough. Furthermore, in Duong et al. (2023), the same behaviour is proven true for $E{mix}(\theta )$ when $t=N-1$. This demonstrates that the qualitative behaviour of the cost function changes significantly when *t* and $\beta$ vary. We conjecture that there exists a critical threshold value of $t^*$ such that: for $t\le t^*$, $E(\theta )$ (where $E(\theta )$ can be either $E_r(\theta )$, $E_p(\theta )$, or $E_{mix}(\theta )$) is always non-deceasing for all $\beta$ when $t\le t^*$, while for $t^*<t\le N-1$, $E(\theta )$ is non-decreasing when $\beta$ is sufficiently small, but is not monotonic when $\beta$ is sufficiently large. Figures 6, A5 suggest that, for small population size *N*, the critical threshold $t^*$ is $N-1$. How to prove this interesting phase transition phenomena for general *N* is elusive to us at the moment and deserves further investigation in the future.

All the simulations in Sect. 5 can be found in the ‘Evolutionary Game Theory’ repository under the ‘Reward and Punishment - General t’ folder.

## Discussion

Over the past decades, there has been a lot of attention given to studying effective incentive mechanisms with the aim of promoting cooperation. Various mechanisms have been tested (Nowak 2006b; Sigmund 2010; Perc et al. 2017; Rand and Nowak 2013; Van Lange et al. 2014), with some of the most efficient ones being institutional incentives, where there is a central decision-maker in charge of applying them. In our model, we adopt this idea of an external decision-maker and entrust them with providing reward, punishment, or hybrid incentives to players interacting via two cooperation dilemmas, the Donation Game or the Public Goods Game. While other works have examined a comparable setting, relatively few have looked at the question of optimising the overall cost to the institution while maintaining a certain degree of cooperation. Moreover, most studies have focused on the full-invest approach (also known as the standard institutional incentive model), in which incentives are always provided regardless of the population composition.

In this paper, we studied the problem of optimising the cost of institutional incentives that are provided conditionally on the number of cooperators in the population (namely, when less than a threshold *t*) while guaranteeing a certain level of cooperation in a well-mixed, finite population of selfish individuals. We use mathematical analysis to derive the cost function as well as the neutral drift and strong selection limits for the case $t = 1$ and the cost function for the case $t = 2$ (using two different initial states). We provide numerical investigation for the aforementioned cases as well as for the others, i.e., for $2 \le t < N-1$. We also introduced cost functions with a general mutation rate for reward, punishment, and hybrid incentives and numerically analysed their behaviour.

For the mathematical analysis of the reward incentive cost function to be possible, we made some assumptions. Firstly, in order to derive the analytical formula for the frequency of cooperation, we assumed a small mutation limit (Rockenbach and Milinski 2007; Nowak et al. 2004; Sigmund 2010). Despite the simplified assumption, this small mutation limit approach has wide applicability to scenarios which go well beyond the strict limit of very small mutation rates (Zisis et al. 2015; Hauert et al. 2007; Sigmund et al. 2010; Rand et al. 2013; Duong et al. 2023). If we were to relax this assumption, the derivation of a closed form for the frequency of cooperation would be intractable. Secondly, we focused on two important cooperation dilemmas, the Donation Game and the Public Goods Game. Both have in common that the difference in average payoffs between a cooperator and a defector does not depend on the population composition. This special property allowed us to simplify the fundamental matrix of the Markov chain to a tri-diagonal form and apply the techniques of matrix analysis (Huang and McColl 1997) to obtain a closed form of its inverse matrix. In games with more complex payoff matrices such as the general prisoner’s dilemma and the collective risk game (Sun et al. 2021), this property no longer holds (e.g., in the former case, the payoff difference, $\Pi _C(i)-\Pi _D(i)$, depends additively on *i*) and the technique in this paper cannot be directly applied. In these scenarios, we might consider other approaches to approximate the inverse matrix, exploiting its block structure.

We intend to utilise analytical techniques to explore the optimisation problems of punishment and hybrid incentives (reward and punishment used concurrently) for the individual-based incentive scheme. This would be interesting because it would allow for a cost comparison between reward or punishment incentives for a certain threshold value *t* and the mixed scheme for the same value of *t*. Finally, there has been little attention given to the use of analysis for obtaining insights into cost-efficient incentives in structured populations or in more complex games (such as the general prisoner’s dilemma and the collective risk game), so this would also be an engaging research avenue.

## Data Availability

Data sharing is not applicable to this article as no datasets were generated or analysed during the current study.

## Appendix

In this appendix, we provide explicit computations of the reward cost function $E_r(\theta )$ for small populations. We also include the calculations of the *W* entries for reward $t = 2$ and some numerical simulations.

### Small Population Examples for Reward

#### $N=3, t=1$

For $N=3, t=1$, we have

$$\begin{aligned} W = \begin{pmatrix} 1&{}-a\\ -d&{}1\\ \end{pmatrix} \end{aligned}$$

and

$$\begin{aligned} W^{-1} = \begin{pmatrix} \frac{-1}{ad-1}&{}\frac{-a}{ad-1}\\ \frac{-d}{ad-1}&{}\frac{-1}{ad-1}\\ \end{pmatrix}. \end{aligned}$$

Thus, the cost function is

$$\begin{aligned} E_r(\theta )&= \frac{N^2\theta }{2} \sum _{j=1}^{1}\frac{(W^{-1})_{1j} + (W^{-1})_{N-1,j}}{N-j} \\&= \frac{9\theta }{4}(W^{-1}_{11}+W^{-1}_{21}) \\&= \frac{9\theta }{4}\frac{-d-1}{ad-1} \\&= \frac{9\theta }{4}\Bigg (\frac{1}{1-\frac{1}{(1+e^{-x})(e^y+1)}}+\frac{1}{\Big (1-\frac{1}{(1+e^{-x})(e^y+1)}\Big )(e^y+1)}\Bigg ), \end{aligned}$$

where $$x=\beta (\delta +\theta )$$ and $$y=\beta \delta $$.

#### $N=3, t=2$

For $N = 3$, $t=2$, we have

$$\begin{aligned} W = \begin{pmatrix} 1&{}-a\\ -c&{}1\\ \end{pmatrix} \end{aligned}$$

and

$$\begin{aligned} W^{-1} = \begin{pmatrix} \frac{-1}{ac-1}&{}\frac{-a}{ac-1}\\ \frac{-c}{ac-1}&{}\frac{-1}{ac-1}\\ \end{pmatrix}. \end{aligned}$$

Hence, the cost function is

$$\begin{aligned} E_r(\theta )&= \frac{N^2\theta }{2} \sum _{j=1}^{2}\frac{(W^{-1})_{1j} + (W^{-1})_{N-1,j}}{N-j} \\&= \frac{N^2\theta }{2}\big [\frac{(W^{-1}_{11}+W^{-1}_{21})}{2}+(W^{-1}_{12}+W^{-1}_{22})\big ] \\&= \frac{9\theta }{4}\Big (\frac{-c-1}{ac-1} \Big ) + \frac{9\theta }{2}\Big (\frac{-a-1}{ac-1}\Big ) \\&= \frac{9\theta }{4}\Bigg (\frac{1}{1-\frac{1}{(1+e^{-x})(1+e^x)}} + \frac{1}{\Big (1-\frac{1}{(1+e^{-x})(1+e^x)}\Big )(1+e^{x})}\Bigg ) \\ {}&\qquad + \frac{9\theta }{2}\Bigg (\frac{1}{1-\frac{1}{(1+e^{-x})(1+e^x)}} + \frac{1}{\Big (1-\frac{1}{(1+e^{-x})(1+e^x)}\Big )(1+e^{-x})}\Bigg ), \end{aligned}$$

where $$x=\beta (\delta +\theta )$$ and $$y=\beta \delta $$.

#### $N=4$, $t=1$

For $N=4$, $t=1$, we have

$$\begin{aligned} W = \begin{pmatrix} 1&{}-a&{}0\\ -d&{}1&{}-b\\ 0&{}-d&{}1\\ \end{pmatrix} \end{aligned}$$

and

$$\begin{aligned} W^{-1} = \begin{pmatrix} \frac{bd-1}{ad+bd-1}&{}\frac{-a}{ad+bd-1}&{}\frac{-ab}{ad+bd-1}\\ \frac{-d}{ad+bd-1}&{}\frac{-1}{ad+bd-1}&{}-\frac{-b}{ad+bd-1}\\ \frac{-d^2}{ad+bd-1}&{}\frac{-d}{ad+bd-1}&{}\frac{ad-1}{ad+bd-1}\\ \end{pmatrix}. \end{aligned}$$

Thus, the cost function is

$$\begin{aligned} E_r(\theta )&= \frac{N^2\theta }{2} \sum _{j=1}^{1}\frac{(W^{-1})_{1j} + (W^{-1})_{N-1,j}}{N-j} \\&= \frac{8\theta }{3}(W^{-1}_{11}+W^{-1}_{31}) \\&= \frac{8\theta }{3}\frac{-d^2+bd-1}{ad+bd-1} \\&= \frac{8\theta }{3}\Bigg (\frac{-1+\frac{1}{(1+e^{-y})(e^y+1)}}{-1+\frac{1}{(1+e^{-y})(e^y+1)}+\frac{1}{(1+e^{-x})(e^y+1)}}\\&\quad - \frac{1}{(e^y+1)^2\Big (-1+\frac{1}{(1+e^{-y})(e^y+1)}+\frac{1}{(1+e^{-x})(e^y+1)}\Big )} \Bigg ), \end{aligned}$$

where $$x=\beta (\delta +\theta )$$ and $$y=\beta \delta $$.

#### $N=4$, $t=2$

For $N=4$, $t=2$, we have

$$\begin{aligned} W = \begin{pmatrix} 1&{}-a&{}0\\ -c&{}1&{}-a\\ 0&{}-d&{}1\\ \end{pmatrix} \end{aligned}$$

and

$$\begin{aligned} W^{-1} = \begin{pmatrix} \frac{ad-1}{ac+ad-1}&{}\frac{-a}{ac+ad-1}&{}\frac{-a^2}{ac+ad-1}\\ \frac{-c}{ac+ad-1}&{}\frac{-1}{ac+ad-1}&{}\frac{-a}{ac+ad-1}\\ \frac{-cd}{ac+ad-1}&{}\frac{-d}{ac+ad-1}&{}\frac{ac-1}{ac+ad-1}\\ \end{pmatrix}. \end{aligned}$$

Thereby, the cost function is

$$\begin{aligned} E_r(\theta )&= \frac{N^2\theta }{2} \sum _{j=1}^{2}\frac{(W^{-1})_{1j} + (W^{-1})_{N-1,j}}{N-j} \\&= 8\theta \Bigg [\frac{(W^{-1}_{11}+W^{-1}_{31})}{3}+\frac{(W^{-1}_{12}+W^{-1}_{32})}{2}\Bigg ] \\&= \frac{8\theta }{3}\Big (\frac{-cd+ad-1}{ac+ad-1} \Big ) + 4\theta \Big (\frac{-a-d}{ac+ad-1}\Big ) \\&= \frac{8\theta }{3}\Bigg (\frac{-1+\frac{1}{(1+e^{-x})(e^x+1)}}{-1+\frac{1}{(1+e^{-x})(e^y+1)}+\frac{1}{(1+e^{-x})(e^x+1)}}\\&\quad - \frac{1}{(e^x+1)(e^y+1)\Big (-1+\frac{1}{(1+e^{-x})(e^y+1)}+\frac{1}{(1+e^{-x})(e^x+1)}\Big )} \Bigg ) + \\ {}&\qquad 4\theta \Bigg (\frac{1}{(e^y+1)\Big (1-\frac{1}{(1+e^{-x})(e^y+1)}-\frac{1}{(1+e^{-x})(e^x+1)}\Big )}\\&\quad - \frac{1}{(1+e^{-x})\Big (-1+\frac{1}{(1+e^{-x})(e^y+1)}+\frac{1}{(1+e^{-x})(e^x+1)}\Big )} \Bigg ), \end{aligned}$$

where $$x=\beta (\delta +\theta )$$ and $$y=\beta \delta $$.

#### $N=4$, $t=3$

For $N=4$, $t=3$, we have

$$\begin{aligned} W = \begin{pmatrix} 1&{}-a&{}0\\ -c&{}1&{}-a\\ 0&{}-c&{}1\\ \end{pmatrix} \end{aligned}$$

and

$$\begin{aligned} W^{-1} = \begin{pmatrix} \frac{ac-1}{2ac-1}&{}\frac{-a}{2ac-1}&{}\frac{-a^2}{2ac-1}\\ \frac{-c}{2ac-1}&{}\frac{-1}{2ac-1}&{}\frac{-a}{2ac-1}\\ \frac{-c^2}{2ac-1}&{}\frac{-c}{2ac-1}&{}\frac{ac-1}{2ac-1}\\ \end{pmatrix}. \end{aligned}$$

Thus, the cost function is

$$\begin{aligned} E_r(\theta )&= \frac{N^2\theta }{2} \sum _{j=1}^{3}\frac{(W^{-1})_{1j} + (W^{-1})_{N-1,j}}{N-j} \\&= \frac{N^2\theta }{2}\Bigg [\frac{(W^{-1}_{11}+W^{-1}_{31})}{3}+\frac{(W^{-1}_{12}+W^{-1}_{32})}{2} + (W^{-1}_{13}+W^{-1}_{33})\Bigg ] \\&= \frac{8\theta }{3}\Big (\frac{c^2+ac-1}{2ac-1} \Big ) + 4\theta \Big (\frac{-a+c}{2ac-1}\Big ) + 8\theta \Big (\frac{-a^2+ac-1}{2ac-1}\Big ) \\&= \frac{8\theta }{3}\Bigg (\frac{-1+\frac{1}{(1+e^{-x})(e^x+1)}}{-1+\frac{2}{(1+e^{-x})(e^x+1)}} - \frac{1}{\Big (1-\frac{2}{(1+e^{-x})(e^x+1)}\Big )(1+e^x)^2} \Bigg ) + \\ {}&\qquad 4\theta \Bigg (\frac{1}{\Big (1-\frac{2}{(1+e^{-x})(e^x+1)}\Big )(e^x+1)} - \frac{1}{\Big (-1+\frac{2}{(1+e^{-x})(e^x+1)}\Big )(e^{-x}+1)}\Bigg ) + \\ {}&\qquad 8\theta \Bigg (\frac{-1+\frac{1}{(1+e^{-x})(e^x+1)}}{-1+\frac{2}{(1+e^{-x})(e^x+1)}} - \frac{1}{\Big (-1+\frac{2}{(1+e^{-x})(e^x+1)}\Big )(1+e^{-x})^2} \Bigg ), \end{aligned}$$

where $$x=\beta (\delta +\theta )$$ and $$y=\beta \delta $$.

### The *W* Entries for $E_r(\theta )$ with $t = 2$

$$\begin{aligned} (W^{-1}_{1,1})&=\frac{1}{1-(-c)(-a)\frac{y_3}{y_2}} \\&= \frac{1}{1-ac\frac{y_3}{y_2}},\\ (W^{-1})_{N-1,1}&=(-1)^{N-2}\left( \prod _{k=1}^{N-2} a_{k+1}\right) \frac{y_{N}}{y_{2}} \phi _{1,1} \\&= (-1)^{N-2} \Bigg (\prod _{k=1}^{N-2} a_{k+1}\Bigg )\frac{1}{y_{2}} \frac{1}{1-ac\frac{y_3}{y_2}} \\&=(-1)^{N^{-2}}\left( a_{2} \cdot a_{3} \cdot \ldots \cdot a_{N-1}\right) \frac{1}{y_{2}} \frac{1}{1-a_{c} \frac{y_{3}}{y_{2}}} \\ \\&=(-1)^{N-2}(-c) \cdot (-d) \cdot \ldots \cdot (-d) \frac{1}{y_{2}} \frac{1}{1-a c \frac{y_{3}}{y_{2}}} \\ \\&=(-1)^{N-2}(-c)(-d)^{N-3} \frac{1}{y_2} \frac{1}{1-a c \frac{y_{3}}{y_{2}}} \\&=c d^{N-3} \frac{1}{y_{2}} \frac{1}{1-a c \frac{y_{3}}{y_{2}}},\\ (W^{-1})_{1,2}&=(-1)^{1}\left( \prod _{k=1}^{1} c_{2-k}\right) \frac{z_{0}}{z_{1}} \phi _{2,2} \\&=-\left( c_{1}\right) \frac{1}{1} \phi _{2,2} \\&=a \phi _{2,2} \\&=a \frac{1}{1-(-c)(-a) \frac{z_{0}}{z_{1}}-(-d)(-a) \frac{y_{4}}{y_{3}}} \\&=a \frac{1}{1-a c-a d\frac{y_4}{y_3}},\\ (W^{-1})_{N-1,2}&=(-1)^{N-3}\left( \prod _{k=1}^{N-3} a_{2+k}\right) \frac{y_{N}}{y_{3}} \phi _{2,2} \\&=(-1)^{N-3}\left( a_{3} \cdot a_{4} \ldots \cdot a_{N-1}\right) \frac{1}{y_{3}} \frac{1}{1-a c-a d \frac{y_{4}}{y_{3}}} \\&=(-1)^{N-3}(-d)^{N-3} \frac{1}{y_{3}} \frac{1}{1-a c-a d \frac{y_{4}}{y_{3}}} \\ \\&=d^{N-3}\frac{1}{y_{3}} \frac{1}{1-a c-a d \frac{y_4}{y_3}}. \end{aligned}$$

### Behaviour of the Cost Functions Under Small Mutation with Different Initial States

In this subsection, we present the results of our numerical simulation in the case of reward incentives for PGG and in the cases of punishment and hybrid incentives for DG, for the two initial starting states.
